# Non-BRAF Mutant Melanoma: Molecular Features and Therapeutical Implications

**DOI:** 10.3389/fmolb.2020.00172

**Published:** 2020-07-24

**Authors:** Irene Vanni, Enrica Teresa Tanda, Bruna Dalmasso, Lorenza Pastorino, Virginia Andreotti, William Bruno, Andrea Boutros, Francesco Spagnolo, Paola Ghiorzo

**Affiliations:** ^1^Genetics of Rare Cancers, IRCCS Ospedale Policlinico San Martino, Genova, Italy; ^2^Genetics of Rare Cancers, Department of Internal Medicine and Medical Specialties, University of Genoa, Genova, Italy; ^3^Medical Oncology, IRCCS Ospedale Policlinico San Martino, Genova, Italy

**Keywords:** melanoma, non-*BRAF* mutation, targeted therapy, driver mutations, genetic, heterogeneity, WES, WGS

## Abstract

Melanoma is one of the most aggressive tumors of the skin, and its incidence is growing worldwide. Historically considered a drug resistant disease, since 2011 the therapeutic landscape of melanoma has radically changed. Indeed, the improved knowledge of the immune system and its interactions with the tumor, and the ever more thorough molecular characterization of the disease, has allowed the development of immunotherapy on the one hand, and molecular target therapies on the other. The increased availability of more performing technologies like Next-Generation Sequencing (NGS), and the availability of increasingly large genetic panels, allows the identification of several potential therapeutic targets. In light of this, numerous clinical and preclinical trials are ongoing, to identify new molecular targets. Here, we review the landscape of mutated non-*BRAF* skin melanoma, in light of recent data deriving from Whole-Exome Sequencing (WES) or Whole-Genome Sequencing (WGS) studies on melanoma cohorts for which information on the mutation rate of each gene was available, for a total of 10 NGS studies and 992 samples, focusing on available, or in experimentation, targeted therapies beyond those targeting mutated BRAF. Namely, we describe 33 established and candidate driver genes altered with frequency greater than 1.5%, and the current status of targeted therapy for each gene. Only 1.1% of the samples showed no coding mutations, whereas 30% showed at least one mutation in the *RAS* genes (mostly *NRAS*) and 70% showed mutations outside of the *RAS* genes, suggesting potential new roads for targeted therapy. Ongoing clinical trials are available for 33.3% of the most frequently altered genes.

## Introduction

Cutaneous melanoma is one of the most aggressive malignancies of the skin. Its incidence is globally growing partly due to the increase of early diagnoses, and contextually, the prevalence is also increasing (Bray et al., 2018; Schadendorf et al., 2018). Until 10 years ago, advanced melanoma was associated with poor survival due to the lack of durable responses to conventional chemotherapy and biochemotherapy (Korn et al., 2008), with a median Overall Survival (OS) of about 6 month in patients with stage IV melanoma. Since 2011, however, the rules of the treatment of stage IV melanoma have been completely rewritten, with the introduction of targeted therapies with BRAF and MEK inhibitors (Larkin et al., 2014; Long et al., 2014; Robert et al., 2016), and immunotherapy with the anti CTLA-4 ipilimumab (Hodi et al., 2010) and the anti-PD-1 nivolumab (Robert et al., 2015) and pembrolizumab (Schachter et al., 2017). These new therapeutic approaches improved melanoma prognosis, resulting in a 5-year survival rate of 34–43% (Hamid et al., 2019; Robert et al., 2019). However, mainly because of primary and acquired resistance to treatments, the majority of patients will ultimately relapse, and only patients harboring a *BRAF* mutation, observed in about 50% of cutaneous melanoma, can receive a targeted treatment with BRAF and MEK inhibitors (Spagnolo et al., 2015). The current state of molecular-target drugs and the current therapeutic scenario for patients with BRAF mutated melanoma, from the introduction of BRAF inhibitors as single agents to modern clinical practice, has been extensively described in a related minireview (Tanda et al., 2020). With the purpose of further improving the prognosis of melanoma patients, several preclinical and clinical trials are studying new actionable mechanisms and/or molecules, to simultaneously tackle multiple resistance mechanisms.

The aim of this review is to describe the landscape of mutated non-*BRAF* melanoma, in light of recent data deriving from Next-Generation Sequencing (NGS) (or Massive Parallel Sequencing – MPS) analysis, focusing on available, or in experimentation, targeted therapies. The advent of MPS, allowing the simultaneous analysis of several genes, led, in the past two decades, to Whole-Exome Sequencing (WES) and Whole-Genome Sequencing (WGS) studies that found several mutated genes in human cancers. The evolution of molecular testing in melanoma, as well as the main techniques and MPS platforms currently in use for *BRAF* mutation testing, have been recently reviewed (Vanni et al., 2020).

The first actionable mutation to be targeted by specific drugs in melanoma, *BRAF* V600, was found in 2002 along several other drivers of human cancers (Davies et al., 2002). Since then, several other genes have been identified as putative drivers of melanomagenesis and/or melanoma progression, and additional candidate drivers are currently being assessed, prompting pharmacogenomics studies on potentially actionable targets (Priestley et al., 2019). However, melanoma is one of the tumors with the highest mutation burden, and results from different studies were frequently not overlapping, possibly due to dissimilar sample size and cohort characteristics (Berger et al., 2012; Hodis et al., 2012; Krauthammer et al., 2012; Snyder et al., 2014; Van Allen et al., 2015). Although this high mutational burden is one of the reason behind the success of immunotherapy in this tumor, it makes it hard to clearly identify novel driver genes that could be used for targeted therapies (Davis et al., 2018).

In 2015, The Cancer Genome Atlas analyzed 333 cutaneous melanoma samples by integrating integrated multi-level genomic analyses, namely WES and low-pass WGS, transcriptome sequencing including miRNA, protein expression, and classified melanoma in four major molecular subtypes: mutant *BRAF*, mutant *RAS*, mutant *NF1* and triple wild-type (The Cancer Genome Atlas Network, 2015). However, as *NF1* mutations can be found in melanomas with concurrent *BRAF* or *NRAS* hotspot mutation, a three-group classification of melanoma (mutant *BRAF*, mutant *RAS*, non-*BRAF*^mut^ /non-*NRAS*^mut^) has been proposed (Palmieri et al., 2018). Although providing an unprecedented insight into the complex mutational spectrum of melanoma, the TCGA study cohort did not include acral and mucosal melanomas. Two years later, this issue was addressed with the molecular characterization of 183 melanoma samples through WGS, including the acral and mucosal subtypes (Hayward et al., 2017). Recently, a joint effort by the TCGA and the ICGC resulted in the description of the molecular spectrum of the largest whole genome dataset of 38 different tumor types, which included a subset of 118 melanoma samples previously described (ICGC/TCGA Pan-Cancer Analysis of Whole Genomes Consortium, 2020).

**FIGURE 1:**
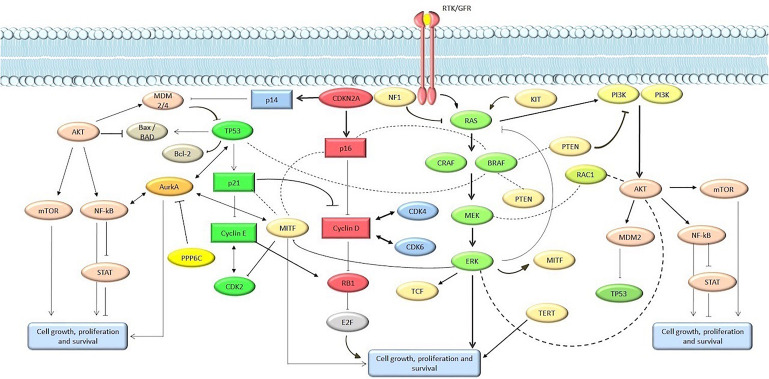
Main pathways involved in melanomagenesis. Genes and proteins are marked in circle and rectangles, respectively.

The spectrum of genomic alterations in melanoma involve multiple genes and signaling networks, but the most frequently altered pathways are MAPK, PIK3CA, KIT signaling, and apoptosis/cell senescence pathways (Figure 1). This review focuses on cutaneous melanoma, including the acral melanoma. To obtain an overview of molecular alterations in skin melanoma, we focused our analysis on all WES or WGS studies on melanoma cohorts or pan-cancer cohorts that included melanoma, for which information on the mutation rate of each gene was available. For selecting TCGA melanoma samples we considered the group’s pan-cancer flagship paper (Hoadley et al., 2018), which included the original cohort of the melanoma-only TCGA study, plus an additional set of 60 skin melanoma samples. With these criteria we collected and combined mutational data from 10 studies published from 2012 to 2019, available from either the cBioportal repository (cBioPortal for Cancer Genomics, 2020) or from the tables within the published manuscripts (Hayward et al., 2017; Birkeland et al., 2018; Kontogianni et al., 2018; Wilmott et al., 2019), to obtain cumulative mutational frequencies of all genes assessed by these studies (Table 1) (Berger et al., 2012; Hodis et al., 2012; Krauthammer et al., 2012; Snyder et al., 2014; Van Allen et al., 2015; Hayward et al., 2017; Kontogianni et al., 2018; Wilmott et al., 2019; cBioPortal for Cancer Genomics, 2020). When present, we filtered out uveal and mucosal melanoma samples, as well as synonymous variants. Moreover, only three studies used for this review were performed with WGS (Berger et al., 2012; Hodis et al., 2012; Hayward et al., 2017; Wilmott et al., 2019), while all the others are WES studies. Non-coding regions were not considered for our analysis, except for *TERT* promoter, whose frequency was calculated on data from a single WGS study (Hayward et al., 2017). Similarly, we used the WGS subset to assess Copy Number Variations (CNVs). An overview of the studies analyzed for this review is found in Table 1.

**TABLE 1:** Skin melanomas samples from the 10 NGS-based studies evaluated for the mutational and CNV frequency in the present report.

	Cutaneous melanomas		Acral melanoma		Total samples (N°)	Reference (PUBMED ID)

	Primary	Metastatic	Primary	Metastatic		
Berger MF, Nature 2012	0	25	0	0	25	22622578
Snyder A, NEJM 2014	0	44	0	5	49	25409260
Van Allen EM, Science 2015	0	92	0	0	92	26359337
Hodis E, Cell 2012	15	85	2	3	100	22817889
TCGA, PanCancer Atlas*	0	363	0	0	363	26091043
Krauthammer M, Nat Genet 2012	35	62	8	9	114	22842228
Hayward NK, Nature 2017	54	86	14	21	175	28467829
Kontogianni G, Cancers 2018	9	0	0	0	9	29596374
Birkeland E, Nat Commun 2018	0	37	0	3	40	29991680
Wilmott JS, Int J Cancer 2019	2	23	0	0	25	30178487
Total samples (N°)	100	822	24	41	992	

*Samples data from TCGA, PanCancer Atlas, were assessed by cBioportal repository (cBioPortal for Cancer Genomics, 2020).

**FIGURE 2:**
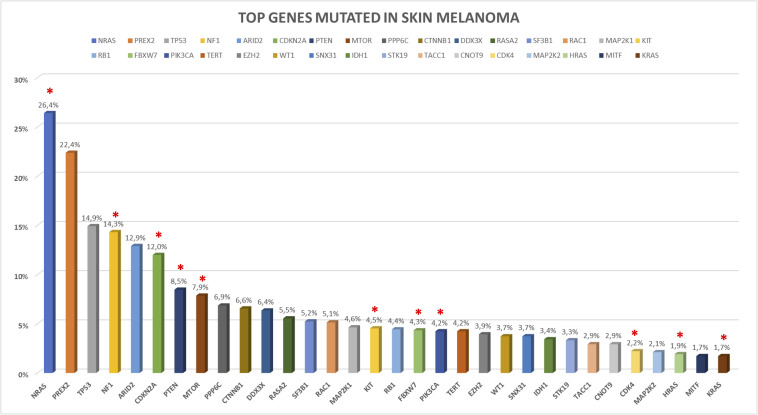
Somatic mutations frequency in top melanoma driver cancer genes based on 992 skin melanoma samples derived by the 10 selected NGS studies. Somatic coding mutations were considered for the analysis excluding synonymous variants. Red asterisks indicate the presence of clinical trials for the altered gene.

**FIGURE 3:**
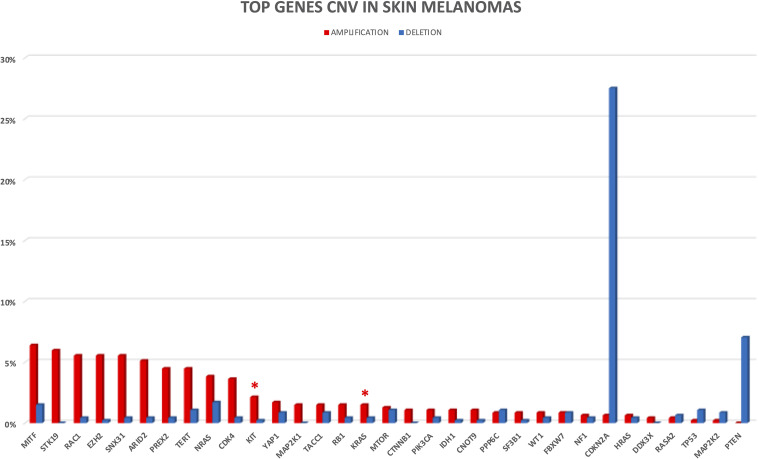
CNV frequency in top melanoma driver cancer genes. Amplifications and deletions are reported in red and blue, respectively. Only four studies with available CNV information were considered for CNV analysis. Red asterisks indicate the presence of clinical trials for the altered gene.

In the following sections, we provide a description of 33 selected established and candidate melanoma driver genes, as well as the mutational and CNV frequency of each gene (Figures 2, 3) in the melanoma samples analyzed, and we describe available, or in experimentation, targeted therapies for each gene/pathway, excluding immunotherapy. Mutational frequency of these genes in melanoma across each study included in the review is displayed in Supplementary Figures S1–S10; ongoing targeted clinical trials are displayed in Table 2.

**TABLE 2:** Clinical trials.

NCT	Phase	*N*	Random	Drugs	Patient selection	Recruiting state	Results

*RAS*							
NCT03979651	Ib/II	29	NO	Trametinib plus hydroxychloroquine	activating *NRAS* mutation	Recruiting	–
NCT03973151	I/II	54	NO	HL-085 (MEK inhibitor)	*NRAS* mutation	Recruiting	–
NCT01763164	III	402	YES	MEK162 vs. dacarbazine	*NRAS* Q61 mutation	Completed	Dummer et al., 2017
NCT01320085	II	183	NO	MEK162	*BRAF V600* or *NRAS* Mutations	Active, not recruiting	Ascierto et al., 2013
NCT01693068	II	194	YES	Pimasertib vs. dacarbazine	*NRAS* mutation	Completed	Lebbe et al., 2016
NCT03932253	I	37	NO	FCN-159	*NRAS* mutation	Recruiting	–
NCT01781572	Ib/II	102	NO	LEE011 + MEK162	*NRAS* mutation	Completed	Sosman et al., 2014
NCT04109456	I	52	NO	IN10018/IN10018 + Cobimetinib	uveal melanoma (UM); *NRAS* mutation	Recruiting	–
NCT02974725	Ib	195	NO	LXH254 + LTT462/Trametinib/Ribociclib	*NRAS* mutation	Recruiting	–
NCT00866177	II	167	NO	AZD6244 (Selumetinib)	V600E or V600K *BRAF* mutation; *NRAS* mutation at codons 12, 13, or 61	Completed	Catalanotti et al., 2013
NCT00338130	II	239	YES	AZD6244/Temodal	*BRAF* and/or *NRAS* mutation	Completed	Kirkwood et al., 2012
NCT03415126	I	49	NO	ASN007	*BRAF* mutation or fusion/*NRAS* and *HRAS* mutation/*MEK1* mutation	Active, not recruiting	–
NCT03989115	Ib/II	144	NO	RMC-4630+ Cobimetinib	*KRAS* mutations and amplifications, *BRAF* Class 3 mutations, or *NF1 LOF* mutations	Recruiting	–
NCT02065063	I/II	28	NO	Trametinib + Palbociclib	*BRAF* V600 wild type and either *NRAS* wild type or *NRAS* mutation type	Completed	–
NCT03634982	I	240	NO	RMC-4630	RTK mutations, amplifications or rearrangements, *KRASG12*, *BRAF* Class 3, or NF1 LOF mutations	Recruiting	–
NCT04284774	II	49	NO	Tipifarnib	*HRAS* genomic alterations	Not yet recruiting	–
NCT01941927	II	20	NO	Trametinib + GSK2141795 (AKTi)	*NRAS* mutation or *NRAS* Wild-type/*BRAF* Wild-type.	Completed	Algazi et al., 2018
NCT04059224	II	58	no	Trametinib	*BRAF* V600 wild-type/*NRAS* wild-type/*NRAS* mutation	Recruiting	–
NCT04198818	I/II	150	no	HH2710	*RAS/RAF/MEK/ERK* mutation	Recruiting	–
NCT02857270	I	272	no	LY3214996/LY3214996 + Midazolam/Abemaciclib/Nab-paclitaxel + Gemcitabine/Encorafenib + Cetuximab/	Activating MAPK pathway alteration, *BRAF* mutation, *NRAS* mutation	Recruiting	–
NCT01390818	I	146	NO	Pimasertib (MEKi) + Voxtalisib (PI3K/mTOR)	Genetic alteration in *PTEN, BRAF, KRAS, NRAS, PI3KCA, ERBB1, ERBB2, MET, RET, KIT, GNAQ, GNA11*	Completed	Schram et al., 2018

***NF1***							

NCT02465060	II	6452	NO	Trametinib (MEKi) Defactinib (FAKi)	*NF1* mutation, *NF2* inactivating mutation	Recruiting	–
NCT03634982	I	240	NO	RMC-4630 (SHP2i)	NF1 LOF	Recruiting	–
NCT03989115	Ib/II	144	no	RMC-4630 + Cobimetinib	*KRAS* mutations and amplifications, *BRAF* Class 3 mutations, or *NF1* LOF mutations	Recruiting	–

***CDKN2A/CDK4***							

NCT02478320	II	12	NO	Ilorasertib (Aurora A/B/Ci)	*CDKN2A* deletion or mutation	Active, not recruiting	–
NCT02202200	I/II	40	NO	Palbociclib (CDK4/6i)	*BRAF* V600E/K mutation, CDNKN2A loss and expression of Rb	Unknown	–
NCT03454919	II	60	NO	Palbociclib (CDK4/6i)	Any gene aberrations in cell cycle pathways, including *CDK4* amplification and/or *CCND1* amplification and/or *CDKN2A* loss	Not yet recruiting	–
NCT02671513	I	30	NO	SHR6390 (CDK4/6i)	Any cell cycle pathway abnormality (e.g., *CDK4* amplify and/or *CCND1* amplify and/or CDKN2A loss)	Unknown	–
NCT00835419	II	12	NO	P276-00 (CDK1/4/9i)	cyclin D1 expression	Completed	–
NCT02065063	I/II	28	NO	Trametinib + Palbociclib	*BRAF* V600 wild type and either *NRAS* wild type or *NRAS* mutation type	Completed	–
NCT01037790	II	304	NO	Palbociclib (CDK4/6i)	*CCND1* amplification, *CDK4/6* mutation, *CCND2* amplification OR any other functional alteration at the G1/S checkpoint.	Completed	–
NCT02308020	II	162	NO	Abemaciclib	Any*	Completed	–
NCT01781572	Ib/II	102	NO	LEE011 (ribociclib) + MEK162	*NRAS* mutation	Completed	Sosman et al., 2014
NCT02974725	I	315	NO	LXH254 + LTT462; LXH254 + Trametinib; LXH254 + Ribociclib	*NRAS* mutation	Recruiting	–

***PTEN***							

NCT03131908	I/II	41	NO	GSK2636771 (Pi3Ki) + pembrolizumab	Evidence of PTEN loss	Recruiting	–
NCT01390818	I	146	NO	Pimasertib (MEKi) + Voxtalisib (PI3K/mTORi)	Genetic alteration in *PTEN, BRAF, KRAS, NRAS, PI3KCA, ERBB1, ERBB2, MET, RET, KIT, GNAQ, GNA11*	Completed	Schram et al., 2018

***MAP2K1/MAP2K2***							

NCT01364051	I	19	NO	Cediranib Maleate + Selumetinib	Any*	Active, not recruiting	–
NCT01941927	II	20	no	Trametinib + GSK2141795 (AKTi)	*BRAF* Wild-type and NRAS mutations	Completed	Algazi et al., 2018
NCT00948467	I	51	NO	TAK-733 (MEKi)	Any*	Completed	Adjei et al., 2017
NCT02296112	II	9	NO	Trametinib	*BRAF* mutations in loci other than V600 (*BRAF* non V600 MUT) or *BRAF* fusion	Active, not recruiting	–
NCT02857270	I	272	no	LY3214996/LY3214996 + Midazolam/Abemaciclib/Nab-paclitaxel + Gemcitabine/Encorafenib + Cetuximab/	Activating MAPK pathway alteration, *BRAF* mutations, *NRAS* mutations	Recruiting	–
NCT04198818	I/II	150	no	HH2710	*RAS/RAF/MEK/ERK* mutations	Recruiting	–
NCT04059224	II	58	no	trametinib	*BRAF* V600 wild-type/*NRAS* wild-type/*NRAS* mutant	Recruiting	–

***KIT***							

NCT02501551	II	36	NO	Regorafenib	*KIT* mutations	Recruiting	–
NCT01028222	II	55	NO	Nilotinib/DTIC	*KIT* mutations	Completed	Guo et al., 2017
NCT01395121	II	29	NO	Nilotinib	*KIT* mutations	Completed	–
NCT01168050	II	25	NO	Nilotinib	*KIT* mutations or amplification	Unknown	Carvajal et al., 2015
NCT00470470	II	30	NO	Imatinib mesylate	*KIT* mutations	Completed	Carvajal et al., 2011
NCT00881049	II	1	NO	Imatinib	*KIT* mutations	Completed	–
NCT01782508	II	40	YES	Imatinib	*KIT* mutations	Unknown	–
NCT00577382	II	52	NO	Sunitinib	*KIT* mutations	Completed	Buchbinder et al., 2015
NCT00424515	II	24	NO	Imatinib	*KIT* mutations	Completed	Hodi et al., 2013
NCT00631618	II	12	NO	Sunitinib	*KIT* mutations	Completed	–
NCT01738139	I	96	NO	Ipilimumab + Imatinib	*KIT* mutations	Recruiting	–
NCT00700882	II	81	NO	Dasatinib	*KIT* mutation or amplification	Active, not recruiting	–
NCT01390818	I	146	NO	Pimasertib (MEKi) + Voxtalisib (PI3K/mTORi)	Genetic alteration in *PTEN, BRAF, KRAS, NRAS, PI3KCA, ERBB1, ERBB2, MET, RET, KIT, GNAQ, GNA11*	Completed	Schram et al., 2018
***FBXW7 and RB1***							
NCT02873975	II	50	NO	LY2606368 (prexasertib)	*MYC* amplification, *CCNE1* amplification, *Rb* loss, *FBXW7* mutation, or another genomic abnormality indicative of replicative stress	Active, not recruiting	–
NCT02202200	I/II	40	NO	Palbociclib (CDK4/6i)	BRAF V600E/K mutation, CDNKN2A loss and expression of Rb	Unknown	–

***PI3KCA***							

NCT01449058	Ib	139	NO	BYL719 + MEK162	Any*	Completed	Juric et al., 2014
NCT02646748	I	159	NO	Pembrolizumb + Itacitinib (JAKi − INCB039110; Pembrolizumab + Parsaclisib (Pi3kδi INCB050465)	Any*	Active, not recruiting	–
NCT04282018	I/II	150	no	BGB-10188/BGB-10188 + Zanubrutinib/BGB-10188 + Tislelizumab	Any*	Recruiting	–
NCT01390818	I	146	NO	Pimasertib (MEKi) + Voxtalisib (PI3K/mTOR)	Genetic alteration in *PTEN, BRAF, KRAS, NRAS, PI3KCA, ERBB1, ERBB2, MET, RET, KIT, GNAQ, GNA11*	Completed	Schram et al., 2018

***WT1***							

NCT03311334	Ib/II	84	NO	DSP-7888 + Nivolumab/DSP-7888 + Pembrolizumab	Any*	Recruiting	
NCT02498665	I	24	NO	DSP-7888	Any*	Completed	

***MITF***							

NCT01065467	I	16	NO	LBH589	Any*	Completed	Ibrahim et al., 2016

***MTOR***							

NCT01960829	II	60	NO	Everolimus	*MTOR* mutation	Unknown	
NCT01166126	II	4	NO	Temsirolimus/AZD 6244	*BRAF* mutation	Terminated	
NCT01014351	II	70	NO	Everolimus + paclitaxel + carboplatin	Any	Completed	Hauke et al., 2013
NCT01522820	I	18	NO	Rapamycin + dendritic Cell Vaccine	NY-ESO-1 expression	Completed	
NCT00655655	I	96	NO	Everolimus + Vatalanib	Any	Completed	Zhu et al., 2020
NCT01596140	I	27	NO	Vemurafenib + Everolimus + Temsirolimus	Any	Completed	
NCT02201212	II	30	NO	Everolimus	*TSC1* or *TSC2* mutation or activating *MTOR* mutations	Completed	

*Any: population not selected for mutation. Clinicaltrials.gov accessed May 10, 2020.

**FIGURE 4:**
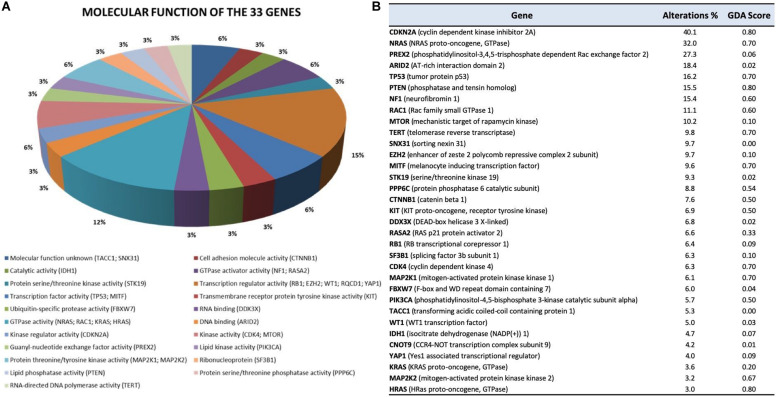
Molecular function **(A)** and ranking **(B)** of the 33 genes analyzed. **(A)** Gene Ontology (GO) molecular function of the 33 genes. GO molecular function was obtained using FunRich v3.1.3, a stand-alone software tool (Pathan et al., 2015). **(B)** The table reports the frequency of genomic alterations (mutations and CNV) resulting from the 10 selected NGS studies, as well as the Gene–Disease Association (GDA) Score obtained from the DisGeNET (Piñero et al., 2015) database using the keyword “melanoma” (C0025202 result). The GDA Score is computed by integrating evidence from multiple sources. The higher the GDA Score, the more reliable the gene–disease relationship is.

Moreover, for each of the 33 genes included in this review and listed here by mutational frequency and/or pathway, we also provide molecular function, frequency alterations (mutations plus CNV) in our dataset, and ranked their effect on melanoma according to the DisGeNET Gene–Disease Association (GDA) Score (Piñero et al., 2015) (Figure 4).

## *RAS* Genes (*NRAS/KRAS/HRAS*)

Ras is a superfamily of small GTPase proteins that regulate cell growth, survival, differentiation and play a key role in transmitting the signal from Receptor Tyrosine Kinases (RTK) to several downstream signaling pathways, in particularly MAPK (mitogen-activated protein kinase) and PI3K (phosphoinositide 3-kinase). The three RAS tissue specific isoforms (*NRAS*, neuroblastoma ras viral oncogene homolog; *KRAS*, Kirsten rat sarcoma viral oncogene homolog; *HRAS*, Harvey rat sarcoma viral oncogene homolog) are frequently mutated in cancers. In cutaneous melanoma, *NRAS* is mutated in about 15–30% of cases, while *KRAS* (∼2%) and *HRAS* (∼1%) play a minor role (Milagre et al., 2010). *NRAS, KRAS*, and *HRAS*, mutations and CNVs in our dataset are reported in Figures 2, 3 and Supplementary Figures S1A–C.

### NRAS

The *NRAS* was the first oncogene identified in melanoma in 1984, and the second most prevalent after *BRAF* (mutation frequency of 30%) (The Cancer Genome Atlas Network, 2015). *NRAS* mutations primarily occur at position 61 (Q61R/K/L accounts for about 80%) and, less frequently, at positions 12 and 13 (G12/13 accounts for about 6%) (Bos, 1989; Lee et al., 2011). These mutations cause an altered GTPase activity that keeps NRAS activated: this always-on signal induces a constitutional activation of the whole MAPK pathway with cell proliferation, dysregulation of the cell cycle and activation of other pro-survival pathways (Hodis et al., 2012).

Melanoma patients harboring mutated *NRAS* display different characteristics compared to those harboring mutated *BRAF*: they are older (>55 years) and have a story of UV exposure, thicker primary tumors and a higher rate of mitosis. Several studies showed that *NRAS* mutations may result in an inferior clinical outcome with a shorter Melanoma-Specific Survival (MSS) (Devitt et al., 2011; Ellerhorst et al., 2011), although partially debated (Omholt et al., 2003; Akslen et al., 2005; Edlundh-Rose et al., 2006; Ugurel et al., 2007; Ellerhorst et al., 2011).

The high number of *NRAS* mutations in cutaneous melanoma did not allow the development of effective drugs: targeting directly *NRAS* remains a great challenge, and the target therapy for *NRAS* mutant melanoma is focused on MEK inhibitors.

### KRAS

*KRAS* encodes for two proteins resulting from alternative splicing of exon 4, KRAS4A and KRAS4B. These proteins have different structures in their C-terminal region and use different mechanisms to localize to cellular membranes (Welman et al., 2000). KRAS4B is the most frequent in human cells and differs from KRAS4A for a C-terminal residue which allows to bind calmodulin and induce its phosphorylation by PKC (Villalonga et al., 2001; Sung et al., 2013). Binding between KRAS4B and calmodulin seems to determine drug resistance, facilitate tumorigenesis and express stem-like markers on the cell surface (Wang et al., 2015). *KRAS* mutations occur most commonly at codon 12 but also at 13 and 61 (Pylayeva-Gupta et al., 2011; Liu et al., 2019). Mutation of G12 interferes with GAP binding and GAP-stimulated GTP hydrolysis and represents ∼12% of all *KRAS* mutations (Tate et al., 2019; COSMIC, 2020). The mutation in G13 decreases GAP binding and its hydrolysis while mutation in codon 61 has a direct role in inhibiting the hydrolysis reaction (Ostrem and Shokat, 2016). *KRAS* mutations are found in 15–20% of cancers, mostly in colorectal and pancreatic adenocarcinomas (Chiosea et al., 2011; Hartman et al., 2012; Zhou et al., 2017; Román et al., 2018). In melanoma, *KRAS* mutations are rare (1.7% of our cases) occurring almost exclusively in codon G12 (Milagre et al., 2010). KRAS mutant remains undruggable.

### HRAS

*HRAS* encodes for a GTPase involved in regulating cell division in response to growth factor stimulation (Wong-Staal et al., 1981). Mutations in *HRAS* cause cell overgrowth and are implicated in a variety of cancers (Rauen, 2007). *HRAS* is altered in 0.97% of all cancers and, rarely, in 1.5% of melanoma (AACR Project Genie Consortium, 2017). Moreover, *HRAS* has been shown to be mutated in Spitz nevi both by CNVs (12/102; 12%) and point mutations (8/12; 67%) (Bastian et al., 1999, 2000). The reason why mutations in *HRAS* lead to Spitz nevi is unclear but could be related to higher affinity for the PI3K-PKB/AKT pathway which would be able to drive the symmetrical overgrowth of cells with an epithelioid morphology without marked activation of the melanizing pathways (Ross et al., 2011). Spitz nevus should not be regarded as a precursor lesion of melanoma. *HRAS* mutation analysis seems to be useful in the differential diagnosis between Spitz nevus and Spitzoid melanoma, and the presence of *HRAS* mutations is a marker of benignity and/or favorable clinical outcome (Dimonitsas et al., 2018).

### RAS Genes – A Clinical Approach

To date, therapeutic inhibition of RAS remains an unmet need. Indeed, there are no approved therapies that specifically target *NRAS*, *KRAS* or *HRAS* and RAS-mutated patients are usually treated with immunotherapy. However, several trials are ongoing (Table 2).

Several years ago *in vitro* studies had already shown that mutated *NRAS* melanoma cells were sensitive to MEK inhibitors (Solit et al., 2006). However, the efficacy of such drugs on mutated *NRAS* cells was lower than that observed in mutated *BRAF* cells, and this lower efficacy could be explained, at least in part, if we consider the complexity of the molecular pathways network involving RAS. From these observations, two hypotheses arose: first, that probably a therapy based on a single drug could be insufficient; second, that the best therapeutic possibility was to target RAS directly (Mandalà et al., 2014). Unfortunately, targeting directly RAS did not give the expected results. Indeed, acting on GTP binding pocket in RAS protein is difficult due to the excessive affinity between RAS and GTP (Baines et al., 2011). In the same way, the inhibition of farnesylation of a cysteine residue, a post translational modification necessary to RAS insertion to the plasma cell membrane, has proven ineffective. Due to these disappointing results, the therapeutic strategies for mutated *NRAS* melanoma reverted on MEK inhibitors.

Initially, the use of MEK inhibitors led to modest results, with an Overall Response Rate (ORR) of 10% and a high incidence of adverse events (Rinehart et al., 2004; LoRusso et al., 2010). Subsequently, the MEK 1/2 inhibitor selumetinib (AZD-6244) was developed (Yeh et al., 2007). Phase I trial enrolled 11 melanoma patients and showed promising results (Adjei et al., 2008, 142886); on this wave, phase II trials comparing selumetinib and chemotherapy (temozolomide and docetaxel) in *BRAF*-WT and *NRAS*-unselected melanoma patients were initiated. The results were unsatisfactory for both trials, with no difference in efficacy outcomes (Kirkwood et al., 2012; Gupta et al., 2014).

Later, other MEK inhibitors were developed. Binimetinib (MEK162) is an allosteric, selective, non-ATP competitive MEK 1/2 inhibitor.

In preclinical studies binimetinib inhibited the growth of *NRAS* and *BRAF* mutated melanoma tumor cells (Winski et al., 2010). In the subsequent phase II trial (Ascierto et al., 2013) MEK162 obtained an ORR of 14.5% and a Disease Control Rate (DCR) of 56%. Median Progression Free Survival (PFS) was 3.6 months, underlying a rapid development of resistance, and the median OS 12.2 months (van Herpen et al., 2014). Phase III trial (NEMO) (Dummer et al., 2016) compared the efficacy of MEK162 versus dacarbazine in 402 *NRAS* mutated, melanoma patients. The ORR was 15% in the binimetinib arm versus 7% in dacarbazine arm. Furthermore, binimetib significantly prolonged median PFS, with 2.8 months [3.9 in patients with normal lactate dehydrogenase (LDH)] versus 1.5 months, respectively [hazard ratio (HR), 0.62]. Interestingly, immunotherapy pretreated patients had a longer median PFS (5.5 months). However, no differences in terms of OS were noted, and binimetinib as single agent did not receive regulatory agencies approval for the treatment of *NRAS*-mutated melanoma.

Finally, pimasertib (PIM; AS703026) has been evaluated in a phase I trial with encouraging results. Phase II study enrolled 194 patients to be treated with pimasertib or dacarbazine. Results showed a significant benefit for pimasertib, with a median PFS of 13.0 versus 6.9 weeks and a DCR of 37.7% versus 26.6%. Unfortunately, no difference in OS was showed (8.9 vs. 10.6 months) (Lebbe et al., 2016).

Combination of MEK inhibition with other targets is currently being evaluated. Among all, the results of a combination trial performed on 14 patients treated with ribociclib (LEE001) and MEK162 are particularly interesting. Indeed, the combination obtained six partial response and six stable disease, with a DCR of 85% (Sosman et al., 2014).

As we mentioned before, NRAS has to undergo some post-translational modifications, like farnesylation (Konstantinopoulos et al., 2007). Farnesyl Transferase Inhibitors (FTIs), like lonafarnib, was developed in an attempt to exploit this phenomenon. FTIs inhibit the function of RAS and seem to be able to sensitizing melanoma cells to RTK inhibitors like sorafenib (Meier et al., 2009). Unfortunately, FTIs failed in clinical trials showing no efficacy against NRAS and KRAS-driven cancers (Smalley and Eisen, 2003; Konstantinopoulos et al., 2007; Niessner et al., 2011; Gajewski et al., 2012; Margolin et al., 2012; Cox et al., 2015). The reason of this failure seems to be due to the action of geranylgeranyltransferase I (GGTase I) in the alternative prenylation (Whyte et al., 1997). FTIs in combination with GGTase I inhibitors have been tested in clinical trials but turned out to be too toxic (Brock et al., 2016). Other approaches attempting to inhibit some post-translational modifications are currently being evaluated. However, the great limit remains the toxicity of these drugs and challenges in delivering siRNA using nanoparticles (Davis et al., 2010).

### PREX2

The *PREX2* gene plays oncogenic roles in human cancers, such as melanoma, since it is involved in PIK3CA-PTEN-AKT signaling pathway (Fine et al., 2009; Srijakotre et al., 2017). It has been demonstrated that ectopic expression of mutant PREX2 accelerates tumor formation of immortalized human melanocytes *in vivo* (Berger et al., 2012). The upregulation of PREX2 and its mRNA increasing expression led to the AKT activation by PTEN phosphorylation and increases tumor proliferation. *PREX2* non-synonymous variants have been found in 44.0% of a 25 patients cohort and in 14.0% of a 107 melanoma samples validation cohort (Berger et al., 2012). Notably, the truncating mutation *PREX2* E824^∗^ was further studied to determine its *in vivo* implications in the context of mutant *NRAS* (Lissanu Deribe, 2016a). *PREX2* truncation E824^∗^ was found to cooperate with *NRAS* mutations but not with *BRAF* V600E mutation, to accelerate melanoma development (Lissanu Deribe, 2016a). In 2017, 100 primary melanoma samples were analyzed by targeted NGS for 35 melanoma-related genes, and *PREX2* mutations were reported in 14 samples (de Unamuno Bustos et al., 2017). Interestingly, recently eighty patients with conjunctival melanoma were examined by WES, identifying three *PREX2* mutations (frequency of 37%) (Demirci et al., 2019). In our skin melanoma dataset, mutations and CNVs in *PREX2* gene are found at frequency of 22.3 and 4.9%, respectively, as shown in Figures 2, 3 and Supplementary Figure S1D.

Although these evidences point to *PREX2* as a key player in melanoma, suggesting that *PREX2* may be a potential therapeutic target, to date no clinical trials are available.

### TP53

The *TP53* tumor suppressor gene, which is universally recognized as the “guardian of the genome,” prevents the cell from dividing and promotes apoptosis. Moreover, it is the most frequently mutated gene in human cancer with a significant prevalence of missense mutations (Hainaut and Pfeifer, 2016). The TCGA database analysis identified across 32 different cancer types 3,786/10,225 (36.8%) patients with *TP53* somatic mutations (Donehower et al., 2019).

Loss of *TP53* was uncommon in thicker, early or in situ melanoma, but is commonly reported as a late event in the development of melanoma (Shain et al., 2015). The Ultra-Violet Radiation (UVR) role on melanoma formation in *BRAF* V600E mice is often associated with mutations in *TP53* (Viros et al., 2014). In our data, mutations and CNVs in the *TP53* gene are present at a frequency of 14.9 and 1.3%, respectively (Figures 2, 3 and Supplementary Figure S2A).

Concerning the use and response of immune checkpoint inhibitor therapy, data are controversial. The *TP53* mutation had previously been considered a potential negative predictor of metastatic melanoma treated with CTLA-4 blockade (Xiao et al., 2018), while a very recent paper showed that cell cycle regulators, such as *TP53* and *CDKN2A*, do not appear to significantly alter clinical outcomes when immune checkpoint inhibitors are used (DeLeon et al., 2020). At the time of the writing of this manuscript, no clinical trial with *TP53*-targeting drugs is ongoing.

### NF1

*NF1* encodes neurofibromin 1, a cytoplasmic protein highly expressed in neurons, Schwann cells, oligodendrocytes, and leukocytes but also involved in RAS pathway as a tumor suppressor through its inhibiting activity as GAP (GTPase-activating protein) that converts the active RAS-GTP to RAS-GDP (Trovó-Marqui and Tajara, 2006). Inactivating variants in NF1, the most frequently mutated gene in melanoma after *BRAF*/*NRAS*/*TP53*, being reported in 10–15% of cases, were described in up to 46% of wild-type *BRAF* and *RAS* melanomas, in particular in male, older or chronically sun-exposed patients, and in copresence of mutations in RASopathy genes, e.g., *PTPN11* and *RASA2*, that enhance its role in melanomagenesis, besides the RAS missing inhibition (Krauthammer et al., 2015). A poorer OS for *NF1*-mutated subtype melanoma has also been described (Cirenajwis et al., 2017). In *BRAF* V600E melanomas, loss of *NF1*, frequently co-occurring with *BRAF* and *RAS* alterations, allows a high level of activity of RAS-GTP and resistance to BRAF inhibitors (Nissan et al., 2014). Since 2000, a role for *NF1* was proposed for the genesis of desmoplastic neurotropic melanoma, an uncommon melanoma with pathologic features in common with schwannomas (Gutmann, 2001; Kiuru and Busam, 2017; Mahalingam, 2017). Moreover, *NF1* together with *BRAF* and *NRAS* has been found significatively mutated in melanoma (Hayward et al., 2017). In our series of skin melanomas, mutations and CNVs are reported in Figures 2, 3 and Supplementary Figure S2B, with a frequency of 14.3 and 1.1%, respectively.

Regarding therapy, a study revealed Calpain1 (CAPN1), a calcium-dependent neutral cysteine protease, as a novel NF1 binding partner that regulates NF1 degradation in melanoma cells. ShRNA-mediated knockdown of CAPN1 or treatment with a CAPN1 inhibitor showed to stabilize NF1 protein levels, downregulate AKT signaling and melanoma cell growth. Moreover, combination treatment of Calpain inhibitor I with MEK inhibitor Trametinib in different melanoma cells seemed to be more effective in reducing melanoma cell growth compared to treatment with Trametinib alone, suggesting that this combination may have a therapeutic potential in melanoma (Alon et al., 2019, p. 1). This novel mechanism for regulating NF1 in melanoma provides a molecular basis for targeting CAPN1 to suppress Ras activation. Despite these data, this novel approach is waiting to be tested within clinical trials. Currently, there are no ongoing clinical trials that evaluate NF1-targeted drugs, but two experimentations regard specifically NF1-mutated melanoma patients, treated with either a MEK inhibitor plus a FAK inhibitor or with RMC-4630, a potent and selective inhibitor of SHP2. The results of these trials have not been published yet (Table 2).

### ARID2

*ARID2* encodes a subunit of the SWI/SNF chromatin remodeling complexes (polybromo- and BGR1-associated factor [PBAF]), which facilitates ligand-dependent transcriptional activation by nuclear receptors. The SWI/SNF multiprotein chromatin remodeling complex is involved in regulating cell growth and proliferation. Mutations in nine genes encoding for subunits of the SWI/SNF chromatin remodeling complex are found in 20.0% of human cancers (Shain and Pollack, 2013). *ARID2* was found frequently mutated in melanoma, with a frequency ranging from 7.0 to 18.0% (Hodis et al., 2012; Krauthammer et al., 2012; Ticha et al., 2019). In our dataset, mutations and CNVs in *ARID2* are found in 12.9 and 5.5%, respectively, as shown in Figures 2, 3 and Supplementary Figure S2C.

To date, studies investigating the biological function of ARID2 in melanocytes and its role as tumor suppressor are missing. In 2018, it has been reported that cancers with inactivating mutations in *ARID2* are more sensitive to PD-1 blockade as well as other forms of immunotherapy (Pan et al., 2018). In a very recent study, a higher sensitivity to different DNA-damaging therapies in ARID2-deficient non-small cell lung cancer cells, likely as a result of the ARID2 involvement in DNA repair, was observed (Moreno et al., 2020). In addition, *ARID2* deficiency showed synthetic lethality with PARP inhibition using veliparib, an inhibitor that has shown good results in the treatment of breast cancer and is included in several clinical trials on breast, ovarian and, lung cancer (Moreno et al., 2020). Overall, recent data prompt further investigation of the role of *ARID2* in melanomagenesis and direct testing of several potential therapeutic compounds already available.

## *CDKN2A/CDK4* Pathway

### CDKN2A

The *CDKN2A* (Cyclin Dependent Kinase Inhibitor 2A) gene encodes two alternatively spliced variants known to function as inhibitors of G1 progression through different mechanisms, e.g., the tumor suppressor p16^INK4A^ and p14^ARF^.

p16^INK4A^ blocks the G1 progression, binding CDK4 and CDK6, and preventing them from interaction with D-cyclins and phosphorylation of RB. p14^ARF^, whose name derives from the presence of an Alternate Reading Frame (ARF) in the transcript, binds MDM2 (Mouse double minute 2 homolog or E3 ubiquitin-protein ligase) whose role is the degradation of p53; therefore, it also plays an indirect role in G1 progression. Furthermore, p14^ARF^ has a role in p53-independent cell functions through the interaction with other proteins (Inoue and Fry, 2018). Loss, e.g., by promoter hypermethylation, or alterations, e.g., homozygous deletions of *CDKN2A*, are described in several tumors. *CDKN2A* is altered in 21.4% of cutaneous melanomas (AACR Project Genie Consortium, 2017). The loss of the *CDKN2A* locus is the most common acquired genetic change in precursor lesions, including in situ melanomas (Shain et al., 2015). Over 75% of cutaneous melanoma metastases have lost one or both alleles of *CDKN2A* (The Cancer Genome Atlas Network, 2015). Recently, a CRISPR-based study (Zeng et al., 2018) demonstrated that *CDKN2A* suppresses the initiation of melanoma invasion through inhibition of *BRN2*, a lineage restricted transcription factor which encodes an established regulator of melanocyte and melanoma invasion (Fane et al., 2017). Then the loss of p16^INK4A^ enhances the motility of melanocytic cells through increased expression of BRN2.

In our dataset, frequencies of *CDKN2A* CNVs and mutations are 28.1% (27.5 and 0.6% for deletion and amplification, respectively) and 12%, respectively (Figures 2, 3 and Supplementary Figure S2D).

### CDK4

*CDK4* (cyclin dependent kinase 4) encodes a member of the Ser/Thr protein kinase family responsible for the phosphorylation and regulation of transcription factors, including RB1, SMAD3, MYC, FOXM1, MEP50, able to mediate cell-cycle progression (Sheppard and McArthur, 2013). p16-cyclinD-CDK4/6-retinoblastoma protein pathway, also known as CDK4 pathway, is dysregulated in 90% of melanomas (Curtin et al., 2005).

*CDK4* amplifications are common in human cancers (Beroukhim et al., 2010). In our dataset, frequency of *CDK4* mutations and CNVs is 2.2 and 4%, respectively (Figures 2, 3 and Supplementary Figure S3A).

### *CDKN2A* and *CDK4*: A Clinical Perspective

To date, several clinical trials are ongoing, attempting to find a way to modulate this pathway (Spagnolo et al., 2015) (Table 2). With regard to *CDKN2A* alterations, drugs on study include: ilorasertib (ABT-348), a potent and ATP-competitive multitargeted kinase inhibitor that inhibits Aurora C, Aurora B, and Aurora A and that suppresses RET tyrosine kinase, PDGFRβ and Flt1; palbociclib and SHR6390, two selective inhibitor of the cyclin-dependent kinases CDK4 and CDK6.

Phase I trial with ilorasertib showed two clinical responses among 58 treated patients, and confirmed a good tolerability and safety profile of the drug (Maitland et al., 2018). Palbociclib, together with abemaciclib and ribociclib, have already been approved for the treatment of metastatic breast cancer, after several studies showing their activity in a spectrum of solid tumors including melanoma. Palbociclib is currently under investigation among patients affected by acral melanoma with documented gene aberrations in cell cycle pathways, including *CDK4* amplification and/or *CCND1* amplification and/or CDKN2A loss. Finally, SHR6390 showed a promising activity in preclinical studies performed on cell lines and human tumor xenograft models.

Considering *CDK4/6* alterations, several drugs are being testing.

P276-00 is a novel inhibitor for CDK-1, CDK4 and CDK9 that has been tested in 16 tumor cell lines from different human cancers, showing a significant antiproliferative effect compared to cisplatin. Interestingly, some cancers showed to be particularly sensitive to P279-00, like tumors of central nervous system, NSCLC, breast cancer and melanoma (Joshi et al., 2007).

Ribociclib also showed some activity in melanomas with activating mutations of *BRAF* or *NRAS*.

A phase Ib/II study with ribociclib plus MEK162 has been performed in 2013. Among 14 NRAS mutant advanced or metastatic melanoma patients, the combinations of drugs allowed to obtain six partial responses and six stable disease (Sosman et al., 2014).

Abemaciclib, another CDK4/6 inhibitor, structurally different from ribociclib and palbociclib, showed to be effective among several human tumors, including melanoma, in preclinical models (Gelbert et al., 2014; Tate et al., 2014). Of note, abemaciclib demonstrated to be particularly effective in BRAF resistant melanoma cells (Yadav et al., 2014). Subsequently, a phase I trial was conducted to evaluate safety and tolerability of abemaciclib and its antitumor activity. A total of 26 melanoma patients were enrolled: one achieved a partial response and the DCR was 27%. Interestingly, abemaciclib was found in the cerebrospinal fluid demonstrating to pass the blood brain barrier and, on this basis, a trial specifically dedicated to patients with brain metastasis was designed. Unfortunately, no information concerning the melanoma cohort are available to date.

### PTEN

The *PTEN* tumor-suppressor gene encodes for a phosphatidyl-inositol-3,4,5-triphosphate 3-phosphatase which negatively regulate the phosphatidylinositol 3-kinase (PI3K)/protein kinase B (AKT) pathway. The *PTEN* gene, which is also known as MMAC1 (mutated in multiple advanced cancers) and TEP1 (TGF-β regulated and epithelial cell-enriched phosphatase) exhibits both protein and lipid phosphatase activities. Therefore, deletion or inactivation of *PTEN* results in constitutive AKT activation. Loss of PTEN function through deletion, mutation, and/or decreased expression, has been found in human sporadic cancers, including melanoma (Bonneau and Longy, 2000). *PTEN* mutations were identified for the first time in 1997 through the analysis of 35 melanoma cell lines (Guldberg et al., 1997). Subsequently, different studies reported that approximately 30% of cutaneous melanoma cell lines harbor *PTEN* mutations or deletions (Guldberg et al., 1997; Tsao et al., 1998) and *in vitro* studies were performed attesting the involvement of *PTEN* LOH in the development of more than 30–40% of melanomas (Robertson et al., 1998). Then, *PTEN* mutations were identified in 4 of 61 (7.0%) metastatic melanoma tumors (Birck et al., 2000), a lower rate compared to previous studies (Guldberg et al., 1997; Tsao et al., 1998), but the authors explained this disagreement due to technical limitations and the *in vitro* selection of cells harboring *PTEN* mutations.

*PTEN* and *NRAS* mutations were described as mutually exclusive (Tsao et al., 2000). Moreover, evidence of cooperation between *BRAF* activating mutations and *PTEN* loss in melanoma development was found, suggesting that the activation of MAPK and AKT pathways may be required for melanoma progression (Tsao et al., 2004).

In conclusion, *PTEN* mutations frequently coexist with *BRAF* mutations, but not with *NRAS*, which can independently activate the PI3K cascade, with a mutation and deletion rate frequency of 8.5 and 7.0% in our dataset (Figures 2, 3 and Supplementary Figure S3B).

To date, no therapeutic strategy specifically targeting PTEN has been developed. Interestingly, some trial evaluating the safety and efficacy of two Pi3K inhibitor, alone or in combination with pembrolizumab, in patients with loss of PTEN, are ongoing (Table 2).

### PPP6C

*PPP6C* gene encodes the catalytic subunit of the PP6 serine/threonine phosphatase complex and regulates cell cycle progression in response to IL2 receptor stimulation (Bastians et al., 1997; Filali et al., 1999). Loss of PPP6C function has been known to cause the increase of Aurora A activity that, as the Aurora A amplification, leads to chromosome instability (Zeng et al., 2010; Hammond et al., 2013). *PPP6C* mutations make melanoma cells susceptible to inhibition by Aurora kinase inhibitors (Gold et al., 2014). Moreover, *PPP6C* mutations in melanoma cells seems to induce an increased autophagy. Indeed, PPP6C mutants bind to the PPP6Rs leading to its rapidly degradation. This increases wild-type PPP6C stability, sensitizing the cells autophagy induction in response to mTORC1 inhibition (Wengrod et al., 2015).

Several NGS studies reported a *PPP6C* mutations frequency around 7% (Gold et al., 2014).

In our dataset, *PPP6C* mutations and CNVs are found in 6.9 and 1.9% of cases, respectively (Figures 2, 3 and Supplementary Figure S3C). In conclusion, *PPP6C* mutations in melanoma may lead to new targeted approaches, such as specific PPP6 inhibitors, but at now, no trials are ongoing.

### CTNNB1

*CTNNB1* gene encodes β-catenin that is a core component of the adherens junctions that play a key role in maintaining of the epithelial cell layers, and in transmitting the contact inhibition signal, by anchoring the cytoskeleton. *CTNNB1* is also part of the Wnt signaling pathway and is involved, e.g., in pre-birth development, the maintenance and renewal of stem cells. Finally, it has a role in the formation of the matrix of hair follicles. Gain-of-function variants of *CTNNB1* cause accumulation of the protein in the nucleus, where it promotes cell proliferation by gene-activation, and have been found in several types of cancer. In melanoma cells, growth is promoted by altering expression of MITF (melanocyte inducing transcription factor) (Widlund et al., 2002), although an opposite effect of invasion-blocking MITF mediated has been observed (Arozarena et al., 2011).

The Wnt pathway may be altered in melanomas by different mechanisms, e.g., somatic variants not only in *CTNNB1* but also in *APC* (adenomatous polyposis coli gene) or *ICAT* (inhibitor of beta-catenin) genes (Reifenberger et al., 2002). However, a major mechanism of promoting melanomagenesis is the cooperation between CTNNB1 and NRAS by suppression of p16^INK4a^ expression, releasing cells from oncogene−induced growth arrest (Curley and Bosenberg, 2008). Furthermore, the CTNNB1 promotes expression, in a positive feedback loop, of Tspan8, which triggers melanoma cell detachment and invasiveness (El Kharbili et al., 2019).

In melanoma, mutations in *CTNNB1* (typically missense mutations localized in exon 3) are relatively infrequent (around 7%) (Hodis et al., 2012; Polakis, 2012; Siroy et al., 2015).

Interestingly, *CTNNB1* (S33C) mutation was found to confer resistance to imatinib in a metastatic melanoma patients harboring *KIT* L576P mutation (Cho et al., 2017). In summary, in our dataset frequency of *CTNNB1* mutations and CNVs is 6.6 and 1%, respectively (Figures 2, 3 and Supplementary Figure S3D). Currently there are no drugs that target beta-catenin right now, nor ongoing clinical trials.

### DDX3X

*DDX3X* gene encodes an ATP-dependent DEAD-box RNA helicase frequently altered in various human cancers, including melanomas (Stransky et al., 2011; Wang L. et al., 2011; Kandoth et al., 2013; Bol et al., 2015; The Cancer Genome Atlas Network, 2015; Ojha et al., 2015; Hayward et al., 2017).

Several studies reported that *DDX3X* is involved in double-stranded RNA unwinding, pre-mRNA splicing, RNA export, transcription, and translation (He et al., 2018; Lin, 2019). Despite its important roles in several cytosolic steps of mRNA metabolism, its function in tumorigenesis remains incompletely known. In our dataset, *DDX3X* mutations and CNVs are found with a frequency of 6.4 and 0.4%, respectively (Figures 2, 3 and Supplementary Figure S4A). Moreover, it has been reported that DDX3X loss decreases MITF mRNA levels, leading to a proliferative-to-metastatic phenotype *in vivo*, and it is implicated in resistance to BRAF inhibitors (Phung et al., 2019).

These studies reveal a *DDX3X* role in melanoma cancer providing a new therapeutic target that should be elucidated in the future.

### RASA2

*RASA2* gene encodes a GTPase-activating protein that suppresses RAS function; therefore mutations or loss of function of RASA2 enhance RAS activation (Yarwood et al., 2006). *RASA2* mutations were initially reported in skin melanoma at a frequency ranging from 2 to 8% by three different NGS studies (Supplementary Figure S4B) (Berger et al., 2012; Hodis et al., 2012; Krauthammer et al., 2012, 1). Overall, frequency of *RASA2* mutations and CNVs in our skin melanomas dataset is 5.5 and 1%, respectively (Figures 2, 3 and Supplementary Figure S4B).

*RASA2* mutations co-occurred in *NF1* mutant melanomas that were BRAF-RAS wild type, in a WES study of 213 melanoma samples (62 cell lines and 151 melanomas); 12.2% (26/212) of melanomas were NF1-mutant/BRAF-RAS-wild-type and nine of them harbored co-mutations in *RASA2* (2 nonsense and 3 R551C) (Krauthammer et al., 2015).

Recently, *RASA2* and *NRAS* mutations were confirmed to be mutually exclusive, with *NF1* mutations significantly co-occurring with *RASA2* mutations in *BRAF* and *NRAS* wild-type melanomas, since RASA2 inhibits NRAS and NF1 inhibits KRAS and HRAS (Arafeh et al., 2019). Indeed, *RASA2-* and *NF1-*mutated genes co-selection in melanoma could be equivalent to oncogenic *RAS* mutation (Arafeh et al., 2019).

To date, clinical trials direct toward RASA2 are not currently available.

### SF3B1

*SF3B1* gene encodes subunit 1 of the splicing factor 3b protein complex, a component of the U2 small nuclear ribonucleoprotein complex (snRNP) that participates in the splicing of pre-mRNAs. Mutations in *SF3B1* can lead to alternative splicing events for multiple genes and are found in several cancers, including uveal (frequency of 10–20%) (Harbour et al., 2013; Martin et al., 2013, p. 1; Dono et al., 2014, p. 1), mucosal (Newell et al., 2019; Nassar and Tan, 2020), and cutaneous melanoma (Kong et al., 2014). *SFRB1* mutations (particularly at codon 625) are rare in cutaneous melanoma despite in uveal, mucosal, leptomeningeal and blue naevi-like cutaneous melanomas are reported (Schilling et al., 2013; Kong et al., 2014). Overall, in our dataset, *SF3B1* mutations and CNVs are 5.2 and 1%, respectively, as reported in Figures 2, 3 and Supplementary Figure S4C.

Very interestingly, *SF3B1* mutations were found to induce cancer cells to produce an abnormal form of the BRD9 RNA molecule, including noncoding DNA sequences or “junk DNA,” which garbled the genetic message (Inoue et al., 2019). This “junk DNA” originated from a viral element that recently inserted itself into the human genome. The use of CRISPR technology and antisense oligonucleotides to suppresses tumors growth deriving by SF3B1-mutant cells opens for future therapeutic approaches, but to date no clinical trials directed to *SF3B1* are available (Inoue et al., 2019).

### RAC1

*RAC1* encodes for a RHO GTPase that plays a key role in cellular cytoskeleton organization and motility and can induce RAS dependent pathways stimulating cell proliferation (Krauthammer et al., 2012, 2015). WES studies led to the discovery of a hotspot mutation (P29S) in *RAC1* gene, defining it as the most frequent driver mutation in sun-exposed melanomas, with a frequency of 5–7% (Hodis et al., 2012; Krauthammer et al., 2012; The Cancer Genome Atlas Network, 2015). The frequency of *RAC1* P29S mutation (overall *RAC1* P29S mutation of 4.1%) was found to be more prevalent in male patients and similar between primary (9.2%) and metastatic tumors (8.6%) (Krauthammer et al., 2012). Indeed, mutations in *RAC1* enhanced melanoma disease progression: *RAC1* P29S analysis in a cohort of 814 primary cutaneous melanomas, with known *BRAF* and *NRAS* mutation status, revealed an association with increased tumor thickness, increased mitotic rate, ulceration, and presence of lymph node metastases at the time of diagnosis (Mar et al., 2014). In general, frequency of *RAC1* mutations and CNVs in our skin melanomas dataset is 5.1 and 5.9%, respectively (Figures 2, 3 and Supplementary Figure S4D).

Activation of RAC1 may be also promoted by mutated *PREX2*. Indeed, truncating mutations in *PREX2* have been shown to activate the GTPase RAC1 abolishing the binding to PTEN and activating the PI3K (phosphatidyl inositol 3 kinase)/Akt signaling pathway (Lissanu Deribe, 2016b; Lissanu Deribe et al., 2016). Concerning treatment, *in vitro* studies have shown that melanoma cell lines harboring the *RAC1* P29S mutation are resistant to BRAF and MEK inhibitors, but its role in conferring this resistance is still to be elucidated (Watson et al., 2014). However, clinical inhibitors of RAC1 are not currently available although SRF/MRTF inhibitors in combination with BRAF inhibitors have been recently demonstrated to have utility in the treatment of BRAF mutant melanoma with an *RAC1* P29S mutation (Lionarons et al., 2019).

High levels of PD-L1 in patients with *RAC1* P29S mutations compared to wild-type *RAC1* melanoma samples from the TCGA cohort were observed (Vu et al., 2015). These findings could open up individualized therapy based on immunological characteristics of patient tumor and the presence of *RAC1* P29S mutations with anti-PD-1 or anti-PD-L1 antibodies treatment in melanoma patients with high expression of PD-L1 harboring *RAC1* P29S mutation.

### MAP2K1/MAP2K2

*MAP2K1*, mitogen-activated protein kinase 1, also known as MEK1, and MAP2K2 (MEK2), mitogen-activated protein kinase 2, encode for mitogen-activated protein (MAP) kinase involved in many cellular processes, such as proliferation, transcription regulation, differentiation and development, and represent the downstream targets of the RAS-RAF-MAPK cascade. Activating mutations in *MAP2K1* and *MAP2K2* represent one of the multiple mechanisms of resistance to BRAF and MEK inhibitors (Spagnolo et al., 2015). Overall *MAP2K1/MAPK2* mutational and CNV prevalence in our skin melanomas dataset is 6.7 and 2.5%, respectively (Figures 2, 3). In particular, melanoma patients have a higher frequency of *MAP2K1* mutations/CNVs compared to *MAP2K2* mutations/CNVs (Figures 2, 3). Notably, the coexistence of *MAP2K1* mutations and *BRAF* or *NRAS* mutations are often observed. In general, *MAP2K1/2* mutations in *BRAF* V600E melanomas are linked to both intrinsic and acquired resistance to BRAF inhibitors.

To date, no clinical trials having MEK1/2-mutated melanoma patients as population of interest are ongoing but, as we mentioned before, MEK inhibitors are being tested in several studies, alone or in combination with other drugs, and their use with BRAF inhibitors has become a standard in the therapy of *BRAF* mutated metastatic melanoma.

Trametinib is a small molecule and a selective MEK1/2 inhibitor, non-ATP competitive.

Based on the encouraging results obtained in phase I (Infante et al., 2012) and phase II trial (Kim et al., 2013, 1), a phase III study (METRIC) with trametinib monotherapy compared to chemotherapy was performed (Flaherty et al., 2012). A total of 322 patients affected by previously untreated *BRAF* V600 E/K stage III or IV melanoma were enrolled. Trametinib was associated with a higher PFS (4.8 months vs. 1.5), a greater OS (6-months OS 81% vs. 67%), and a higher rate of responses (22% vs. 8%). Based on these data, trametinib was approved as single agent by the FDA in May 2013. Despite this, currently MEK inhibitors do not play a role in monotherapy in *NRAS*-mutated or in WT patients but could play a role in combination with immunotherapy or with other target agents.

Finally, TAK-733, a selective MEK1/2 inhibitor, showed a broad antitumor activity in melanoma cell lines and in 10 out of 11 patient-derived xenografts models (Micel et al., 2015). In phase I study, performed among 51 patients, TAK-733 showed a manageable toxicity profile but a limited antitumor activity, with partial responses obtained just in 5% of patients (Table 2) (Adjei et al., 2017).

### KIT

*KIT* encodes for a class III tyrosine kinase receptor that is expressed on several cell types, including melanoblasts and differentiated melanocytes, but also hematopoietic progenitors and mast cells. The binding to the stem cell grow factor causes c-kit homodimerization that leads to the phosphorylation of tyrosine residues by activating the MAPK/ERK and the PI3K/AKT/mTOR pathways (Lennartsson and Rönnstrand, 2012). *KIT* was initially thought to act as a tumor suppressor gene, because its presence in normal melanocytes and benign nevi, and its loss during progression and in metastatic melanoma was reported (Montone et al., 1997; Shen et al., 2003; Isabel Zhu and Fitzpatrick, 2006). Moreover, loss of c-kit expression was observed in different cultured melanoma cells (Lassam and Bickford, 1992; Natali et al., 1992; Zakut et al., 1993) and related to a higher metastatic potential of melanoma xenografts in nude mice (Gutman et al., 1994). However, *KIT* also acts as an oncogene. Indeed, it has been found an increase of *KIT* mutations and/or CNVs in mucosal (39%), acral (36%), and melanomas arose on chronically sun-damaged skin (28%) (Curtin et al., 2006). Interestingly, a recent meta-analysis reported *KIT* mutations in 497 (9.5%) melanoma patients analyzing 5,224 patients from 32 studies selected (Gong et al., 2018). Moreover, a close association with older age, acral, mucosal, or chronic sun-damage sites, but not with any histological features or tumor stage was found (Gong et al., 2018). Approximately 70% of *KIT* mutations identified in melanoma and leading to constitutive activation of kinase activity are localized in exon 11 (L576P) or exon 13 (K642E) (Shtivelman et al., 2014). Mutations in *KIT* are generally mutually exclusive with other driver mutations, including *NRAS* and *BRAF*. In our skin melanomas dataset, *KIT* mutations and CNVs are found in 4.5 and 2.3% of patients, respectively (Figures 2, 3 and Supplementary Figure S5C).

The activity of imatinib mesylate in *KIT* mutated melanoma patients was explored through three single arm phase II trials (Table 2). Carvajal et al. treated 28 patients affected by *KIT* mutated advanced or metastatic melanoma with imatinib 400 mg twice daily (Carvajal et al., 2011). Among the 25 evaluable patients, two complete and four partial responses were observed, with a median time to progression of 12 weeks and a median OS of 10.7 months. Best responses were observed among patients with mutations involving recurrent hotspots or with a mutant – WT allelic ratio superior of 1 (40 vs. 0%). Interestingly, particularly good responses were obtained among patients with K642E mutation, that showed an ORR of 50% and a DCR of 100%.

Guo et al. (2011) reported data from 43 treated patients who received imatinib 400 mg daily or, in case of disease progression, 800 mg daily. The median PFS was 3.5 months with a 6-month PFS of 36.6%, while median OS was 12 months. Globally, the (DCR) was 53.3%: 10 patients (23.3%) and 13 patients (3.2%) achieved partial response and stabilization of disease, respectively. Overall, 18 patients (41.9%) achieved tumor regression. Specifically, 9/10 partial responses were observed in patients with mutations in exons 11 or 13. The overall 1-year survival rate was 51%.

Finally, in a third study published by Hodi et al. (2013), 25 patients affected by metastatic melanoma of the mucosa, acral or chronically UV-damaged skin with amplifications or *KIT* mutations received 400 mg of imatinib daily or, in the absence of a clinical response, twice a day. This study confirmed the clinical activity of imatinib, mainly concerning *KIT* mutations: indeed, all responses were achieved among patients with *KIT* mutations while the best response observed in patients with amplifications was stable disease. OS was 12.5 month in the overall cohort, similar to the previously reported trials.

Other KIT inhibitors showed activity in *KIT* mutated advanced melanomas (Table 2).

Nilotinib is a small molecule that showed activity among *KIT* mutated advanced or metastatic melanoma patients in four clinical studies. In the first trial published in 2015 (Carvajal et al., 2015), 19 patients, mostly in disease progression after treatment with imatinib, achieved an ORR of 15.8% and a median OS of 9.1 months. In a second trial, 39 patients were treated with nilotinib (Lee S. J et al., 2015): one experienced complete response and six partial response; median PFS was 3.3 months and median OS 11.9 months. The subsequent phase II trial (TEAM), an ORR of 26.2% was achieved among 42 patients naïve for previous KIT inhibition (91% of which in the presence of exon 11 mutations), a median PFS of 4.2 months and a median OS of 18 months. In another phase II study, 25 patients were treated with an ORR of 16% at six months of observation, median PFS of 6 months and median OS of 13.2 months (Guo et al., 2017).

Sunitinib is another molecule tested in 12 patients with advanced or metastatic melanoma, and obtained four clinical responses in a small clinical trial (1 complete response and 3 partial response) (Minor et al., 2012).

Finally, dasatinib was evaluated in 22 patients with advanced or metastatic *KIT* mutated melanoma. With regard to clinical activity, four partial responses and seven stable diseases were obtained. The median PFS was 4.7 months and median OS 12.3 months (Kalinsky et al., 2017).

In conclusion, to date it is recommended to test *KIT* mutations (especially exons 11 and 13) in acral, mucosal, and unknown origin melanomas, as well as cutaneous ones arising on chronically damaged skin, to offer an additional therapeutic option.

### RB1

*RB1* acts as a tumor suppressor gene by regulating cell cycle division: when dephosphorylated, it interacts directly with E2F1 and inhibits its transcriptional activity with cell cycle arrest. Thus, the cell cycle is finely regulated by CDKs with CDKIs varying levels of RB phosphorylation, E2F family and TP53; alterations that disrupt the p16INK4A:cyclinD-CDK4/6:RB functional pathway may the first critical step leading to melanomagenesis (Bartkova et al., 1996; Lee B. et al., 2015). Cells deficient or with low levels of RB go into p53-mediated apoptosis: this phenomenon is particularly important to identify new compounds able to activate p53 (Knudsen and Wang, 2010). In mouse melanoma cells Rb1 cooperates with MITF to activate expression of Tyr and Cdkn1a/p21Cip1 (Carreira et al., 2005). *RB1* is also important in maintaining chromatin structure, stabilizing histone methylation (Shao and Robbins, 1995). Summing up, RB is likely a multifunctional protein that binds to at least 100 other proteins (Morris and Dyson, 2001). *RB1* is altered in about 4% of all cancers (MCG)^1^. In our dataset, the most common somatic alterations in *RB1* are mutations with a frequency of 4.4% while CNVs are reported in 1.9% of skin melanoma patients (Figures 2, 3 and Supplementary Figure S5D). To date, no clinical trials specifically designed to target RB are ongoing. However, patients with *RB1* loss are specifically considered in one clinical trial with LY260636, a checkpoint kinase 1 inhibitor. The trial is active, not recruiting.

### FBXW7

*FBXW7* is a critical tumor suppressor gene and a member of the F-box protein family, ubiquitin ligase complex, that controls proteasome-mediated degradation of oncoproteins such as cyclin E, c-Myc, Mcl-1, mTOR, Jun, Notch, and AURKA, STAT2 (Minella and Clurman, 2005; Yeh et al., 2018; Lee et al., 2020). Inactivating mutations in *FBXW7* have been described in a variety of human tumors and cancer cell lines (Akhoondi et al., 2007). Loss of function of *FBXW7* in several human cancers has clinical implications and prognostic value: the use of rapamycin has proven to inhibit breast cancer cells with loss of *FBXW7* by mTOR inhibition (Mao et al., 2008; Yeh et al., 2018).

FBXW7 expression was reduced in primary and metastatic melanoma compared with dysplastic and normal nevi and an increase in FBXW7 expression was significantly correlated with a better 5-year patient survival. *In vitro* studies demonstrated that FBXW7 inhibited human cell migration through MAPK/ERK signaling pathway suggesting its prognostic and potential therapeutic role for melanoma treatment (Cheng et al., 2013). Several WES and WGS studies reported a *FBXW7* mutations in around 4% of melanomas (Wei et al., 2011; Berger et al., 2012; Hodis et al., 2012; Krauthammer et al., 2012; The Cancer Genome Atlas Network, 2015; Hayward et al., 2017; ICGC Data Portal, 2020). WES of 103 cutaneous melanomas, including 77 tumor samples and 26 cell lines, found *FBXW7* mutations (8.1%) independently of *BRAF* or *NRAS* mutation (Aydin et al., 2014). In the same study, the authors discovered that inactivation of *FBXW7* gene determines enhanced tumorigenesis by NOTCH1 activation (Aydin et al., 2014). These evidences open up to *FBXW7* potential therapeutic targeting through modulating NOTCH1 signaling (Aydin et al., 2014). Moreover, another study revealed FBXW7α deficiency leading to HSF1 (Heat shock factor 1) accumulation and subsequent activation of the invasion-supportive transcriptional program and metastatic potential of human melanoma cells (Kourtis et al., 2015).

In our dataset, frequency of *FBXW7* mutations and CNVs is 4.3 and 1.7%, respectively (Figures 2, 3 and Supplementary Figure S6A).

To date, just one clinical trial designed to enroll specifically FBXW7-mutated patients is active (Table 2). LY2606368, the drug on study, performed well in a phase I trial where it was tested in 45 patients affected by solid tumors, who experienced treatment failure with standard therapies. Among 43 evaluable patients, two partial responses and 15 stable diseases were achieved, with a DCR of 37.7% (Hong et al., 2016).

### PIK3CA

*PIK3CA* encodes the protein p110α, the catalytic subunit of phosphatidylinositol 3-kinase (PI3K). PI3K signaling has a role in many cell activities, e.g., cell proliferation, migration, survival.

Alterations in *PIK3CA* have an oncogenic effect mostly related to activating variants in two hotspots located in the regions of helical and kinase domains. The PI3K-AKT pathway plays a significant role in melanomagenesis, frequently by activating RAS-RAF-MEK-ERK pathway, e.g., for *NRAS* activating variants or loss of *PTEN* (Davies, 2012), as demonstrated by studies on resistance to targeted therapies based on BRAF inhibitors (Deng et al., 2012; Penna et al., 2016). Usually, *PIK3CA* activating mutations are rare in melanoma with a frequency of 5%, despite the ability of activated *PIK3CA* (H1047R) to cooperate with *BRAF* V600E promoting melanomagenesis in mouse models (Marsh Durban et al., 2013). *PIK3CA* mutations frequently co-occurred with either a *BRAF* or an *NRAS* mutation (The Cancer Genome Atlas Network, 2015). In our skin melanoma dataset, *PIK3CA* mutations and CNVs are 4.2 and 1.5%, respectively, as shown in Figures 2, 3 and Supplementary Figure S6B.

Several clinical trials with PI3K inhibitors are ongoing (Table 2).

Alpelisib (BYL-719) is an oral selective inhibitor of PI3K isoform-α that showed to be active against the somatic PI3Kα mutations and wild-type PI3Kα (Fritsch et al., 2014). Based on these data, 58 patients with *BRAF* or *RAS* mutated advanced solid tumors were enrolled in a phase Ib trial and were treated with BYL-179 plus MEK162. A total of five *NRAS* mutated patients experienced a partial response (Juric et al., 2014).

Parsaclisib is a novel Pi3kδ inhibitor that exhibited an excellent profile in xenograft models (Yue et al., 2019, 0504). To date, one clinical trial is ongoing to assess the efficacy and the safety of the combination between parsaclisib plus pembrolizumab and itacitinib, a JAK1 inhibitor. The association parsalisib plus itacitinib showed good results in a phase I/II trial for hematological malignancies, with an ORR of 67–78%, respectively in mantle cell lymphoma and marginal zone lymphoma. Lower results were obtained among patients with diffuse large B-cell lymphoma (ORR 30%) (Forero-Torres et al., 2019).

The combination between pimasertib, a MEK1/2 inhibitor, and voxtalisib, a dual PI3K/mTOR inhibitor among patients affected by solid tumors, including melanoma, was tested in a phase Ib trial in 146 patients. In December 2018, data from this experimentation were published, showing a DCR of 52% with one complete response and five partial responses. Unfortunately, the toxicity profile of this combination was considered not acceptable (Schram et al., 2018).

### EZH2

*EZH2* encodes a histone methyltransferase that constitutes the catalytic component of the polycomb repressive complex-2 (PRC2) which has a role in epigenetic silencing during cell differentiation, in particular in the development of the hematopoietic and central nervous systems. EZH2 can also induce an epithelial-to-mesenchymal transition in the cancer cells, increases their metastatic potential (Min et al., 2010) and acts as a coactivator for transcription factors including the androgen receptor (Xu et al., 2012).

More recently it has been described the role of EZH2 as a recruitment platform for DNA methyltransferases in epigenetic repression (Viré et al., 2006; Barsotti et al., 2015; Moran et al., 2018).

The EZH2-dependent expression of genes associated with cell motility contributes to early phases of metastasis (Manning et al., 2015) while activating variants promotes melanoma progression inactivating tumor suppressor genes (Tiffen et al., 2015). The reactivation of tumor suppressors was correlated to increased survival confirming that EZH2-mediated epigenetic repression has a major role in advanced melanoma progression (Zingg et al., 2015). A study suggested the possibility that combined immunohistochemical expression of EZH2, H3K4me2, and H3K27me3 might identify cancer cells with potential stem cell properties; another relevant data that many epigenetic changes are pharmacologically reversible (Kampilafkos et al., 2015; Mahmoud et al., 2016).

*EZH2* mutations and CNVs in our dataset are detailed in Figures 2, 3 and Supplementary Figure S6C, with an overall mutational rate of 3.9 and 5.7%, respectively.

Of note, coexistence of *BRAF* V600E mutation and *EZH2* amplification is rather prevalent in melanoma. Indeed, in a cohort of 138 patients with *BRAF* V600E-mutated melanoma, 40 cases (29.0%) showed EZH2 gain. Moreover, a significant difference in overall survival and disease-free survival between no EZH2 copy number gain and gain groups was reported (Yu et al., 2017).

A recent study highlights that both benign melanocytes and cutaneous melanomas frequently harbor amplifications of *EZH2* that silence genes correlated to the integrity of the primary cilium (Zingg et al., 2018).

Vemurafenib and trametinib induce senescence consistent with downregulation of c-MYC but an EZH2 variant limits this effect (Hartman et al., 2019). EZH2 has a role in differentiation of CD4+ T-cells and in the function of T regulatory cells. Its activation causes immune suppression and it has been suggested that EZH2 inhibitors may have a role in combination with immunotherapy and targeted therapies to prevent immunosuppression (Tiffen et al., 2016). It has been further demonstrated that EZH2 controls melanoma escaping mechanisms during T cell-targeting immunotherapies (Zingg et al., 2017) and because the upregulation of EZH2 and its histone modification H3K27me3 seems correlated to melanoma progression and resistance to immune checkpoint blockade, clinical trials based on EZH2 inhibitors are strongly suggested (Hoffmann et al., 2020).

### WT1

*WT1* encodes a transcription factor implied in the prenatal development of kidneys and gonads, mainly known for its action in cell differentiation and apoptosis. Moreover, *WT1* has a role in tumorigenesis controlling several other genes [e.g., Pecam-1 (CD31) and c-KIT (CD117)], and modulating vascularity, immune response and metastasis formation (Wagner et al., 2014). High expression of WT1 is described in leukemias and in solid tumors and it seems correlated to the chemoresistance and poor outcome. Interestingly, inhibition of cell proliferation by shRNA-WT1, cisplatin, and gemcitabine in B16F10 cells induces cell death and potentiates the action of anticancer drugs by inducing synergistic effects both *in vitro* and *in vivo* (Zapata-Benavides et al., 2019). Although WT1 could be expressed by Spitz naevi and in up to one third of dysplastic naevi, it is considered as a diagnostic tool in melanoma diagnosis (Wilsher and Cheerala, 2007). Indeed, WT1 is expressed in more than 80% of malignant melanoma cells, but not in normal skin or benign melanocytic nevi (Wagner et al., 2008). WT1 protein expression was associated with shorter overall survival in melanoma (Garrido-Ruiz et al., 2010) and deemed as a target antigen for immunotherapy since a novel signaling mechanism mediated by PPARbeta ligands, which led to melanoma cell growth suppression through the direct repression of WT1, was described (Michiels et al., 2010, 1). The silencing of WT1 through shRNAi has a synergistic effect with doxorubicin and cisplatin, sensitizing B16F10 melanoma cells (Zapata-Benavides et al., 2012). *WT1* somatic mutations are described in several cancers, with cutaneous melanoma having the greatest prevalence. Two similarly sized WES analysis of 100 and 114 skin melanomas found *WT1* somatic mutations in the 3 and 1.8% of skin melanoma patients, respectively (Hodis et al., 2012; Krauthammer et al., 2012) (Supplementary Figure S6D). In summary, frequency of *WT1* mutations and CNVs in our skin melanomas dataset are 3.7 and 1.3%, respectively (Figures 2, 3 and Supplementary Figure S6D).

To date, one trial with DSP-7888 plus nivolumab or pembrolizumab in advanced solid tumors including melanoma, is ongoing (Table 2). DSP-7888 is a peptide cancer vaccine that includes WT1 derived peptides: it has shown to induce both a CD8+ and CD4+ mediated immune response against WT1 overexpressing tumor cells.

### SNX31

*SNX31* encodes for the sorting nexins protein involved in membrane trafficking (Worby and Dixon, 2002; Ghai and Collins, 2011; Ghai et al., 2014). It is upregulated in more than 50% of bladder carcinoma transitional cell, but to date its role in cancer is poorly understood. In melanoma, missense mutations in *SNX31* were first reported with a 7% frequency (Hodis et al., 2012). Interestingly, a 9.0% mutation frequency was reported in 46 primary mucosal melanomas (Kim et al., 2017). In our skin melanomas dataset *SNX31* mutations and CNVs are 3.7 and 6.0%, respectively (Figures 2, 3 and Supplementary Figure S7A). No clinical trials with SNX31 inhibitors are ongoing.

### IDH1

*IDH1* encodes for a single, soluble, cytoplasmic isocitrate dehydrogenase 1 enzyme, that converts isocitrate to α-ketoglutarate (also known as 2-oxoglutarate) in an NADP^+^ dependent manner, protecting cells against reactive oxygen species (ROS) and radiations (Minard and McAlister-Henn, 1999; Jo et al., 2002). First described in glioma (Balss et al., 2008, p. 1; Parsons et al., 2008; Yan et al., 2009, p. 2), mutations in *IDH1* and *IDH2* (isocitrate dehydrogenase 2 (NADP+), mitochondrial) were described in several cancers, including melanoma (Lopez et al., 2010). *IDH1* and *IDH2* mutations occur primarily in the catalytic domain at residue R132 in *IDH1*, and R140 and R172 in *IDH2*. The mutant IDH1/IDH2 proteins lead to a reduction of α-ketoglutarate (α-KG) with a relative production of oncometabolite 2-hydroxylglutarate (D-2HG) that it is likely to play a major role in the pathophysiology of tumors blocking cellular differentiation by competitively inhibiting αKG-dependent dioxygenases involved in histone and DNA demethylation (Mondesir et al., 2016). The first *IDH1* mutation in melanoma was reported in 2010 (Lopez et al., 2010). The authors also revealed *BRAF* V600E mutation, but no *NRAS/TP53/CDKN2A/CDKN2B* mutations, in one metastatic sample carrying the R132C mutation, analysing 78 patients. Moreover, *IDH2* R172 mutations were not detected in any of these samples (Lopez et al., 2010). The next year, in a cohort of 142 primary non-epithelial tumors, it was shown that about 10.0% of metastatic melanoma lesions (4/39) harbored an *IDH1* or *IDH2* heterozygous mutation, equally distributed (Shibata et al., 2011). This study confirmed the co-occurrence of *BRAF* mutation with *IDH1* mutation previously observed, and demonstrated that mutant *IDH1* conferred in a *BRAF* V600E melanoma cell line acquired *in vivo* growth activities and enhanced activation of the MAPK and STAT3 pathways (Lopez et al., 2010; Shibata et al., 2011). Mutant *IDH1* also reduced the expression of RASSF1, DHRS1, ADH5, whereas it induced the expression of JUN, MYCN, and ATF3 (Lopez et al., 2010).

In our skin melanoma dataset, mutations of *IDH1* are 3.4% (Figure 2 and Supplementary Figure S7B) while CNVs are 1.3% (Figure 3 and Supplementary Figure S7B).

To date, no FDA-approved drugs or clinical trials are available for melanoma patients carrying *IDH1/IDH2* mutations. Indeed, only two compounds, Enasidenib (AG-221), a DH2-specific inhibitor, and Ivosidenib (AG-120), a IDH1-specific inhibitor, have been approved by FDA for patients with relapsed or refractory acute myeloid leukemia (Stein et al., 2017; DiNardo et al., 2018).

Thanks to these evidences, future clinical studies could be pursued investigating the efficacy of IDH inhibitors for melanoma cancer.

### STK19

*STK19*, also known as *RP1*, encodes a nuclear serine/threonine kinase (Shen et al., 1994; Gomez-Escobar et al., 1998). STK19 protein can bind ATP phosphorylated a-casein proteins at Serine/Threonine residues and histone at Serine residues, therefore phosphorylation of STK19 is involved in transcriptional regulation (Gomez-Escobar et al., 1998). STK19 is important for the transcription-related DNA damage response since it is involved in DNA repair during active transcription and in nuclear signal transduction (Boeing et al., 2016). *STK19* mutations have been found with a frequency of around 4.1% in melanoma, with D89N mutation present at 3.3%, whereas no *SKT19* mutations were detected in nevus associated-melanomas (Hodis et al., 2012; Shitara et al., 2015). In our dataset, *STK19* mutations and amplifications are reported in 3.3 and 6.0% of samples selected (Figures 2, 3 and Supplementary Figure S7C).

In a recent study, STK19 was identified as a novel regulator of NRAS function (Yin et al., 2019). *STK19* alterations were mutually exclusive with *BRAF* (Yin et al., 2019). Through *in vitro* and *in vivo* experiments STK19 was found to phosphorylate the residue S89 of *NRAS* activating NRAS signaling via the MEK-ERK and PI3K pathways. In *NRAS* Q61R transgenic mice the *STK19* D89N mutant promoted oncogenic NRAS-driven melanomagenesis. An STK19 inhibitor (ZT-012-037-1) was able to inhibit NRAS activation in *NRAS-STK19* mutant mice preventing NRAS-driven melanoma development and growth (Yin et al., 2019), revealing a promising therapeutic strategy for NRAS-mutant melanoma tumors treatment (Yin et al., 2019). Based on these last observations, several natural compound libraries were screened using a phosphorylation assay-based approach and chelidonine was identified as a potent and selective inhibitor of STK19, providing a novel option for targeting NRAS-mutant cancers (Qian et al., 2020). Nevertheless, clinical trials are not available.

### MITF

Microphthalmia-associated transcription factor is a basic helix-loop-helix (hHLH)-leucine zipper protein that plays a role in the development of neural crest-derived melanocytes and retinal pigment epithelial cells, and is encoded by *MITF* gene (Fuse et al., 1999). MITF is directly involved in the expression of genes that encode melanin synthesis enzymes (TYR, TYRP1, and DCT), melanosome proteins (PMEL, MLANA, and RAB27A), proteins involved in the cell cycle (CDKN1A, CDKN2A, TBX2, and CDK2) and in cell survival (e.g., BCL2, BIRC7, HIF1A, and MET) (Levy et al., 2006; Cheli et al., 2010; Vachtenheim and Borovanskı, 2010), DNA replication and repair, cell proliferation, and mitosis (Strub et al., 2011; Webster et al., 2014). This well-known role in melanocyte development led MITF nicknaming as the “master regulator” of melanocytes (Levy et al., 2006). Despite MITF acts together with many transcription factors as SOX10, YY1, TFAP2A, LEF1, RB1, IRF4, and PAX3 (Seberg et al., 2017), its activity level determines the phenotype adopted by melanoma cells, whether invasive, proliferative, or differentiated according to the MITF rheostat model (Goding, 2011). *MITF* locus is amplified in 7.9% of skin melanomas, while in our dataset, *MITF* somatic mutations are quite rare, with a frequency of 1.7% (Figures 2, 3 and Supplementary Figure S7D). *MITF* amplification is more prevalent in metastatic melanomas and correlates with decreased overall patient survival. It is responsible for resistance to conventional chemotherapy and BRAF inhibition (Garraway et al., 2005). Recently, Wang et al. provided evidence that p300/CBP inhibition suppressed the melanoma-driven transcription factor, MITF, and could be utilized as a potential therapy for treating melanoma (Wang R. et al., 2018; Kim et al., 2019). To date, no clinical trials are attempting to target MITF directly; on the other hand, MITF-mediated pathways targeting through histone deacetylase inhibitors (HDACi) has been investigated with disappointing results. Indeed, despite HDACi showed the ability to silence MITF promoter *in vitro* and *in vivo* (Yokoyama et al., 2008), this ability was not observed among treated patients in a phase I trial performed between 2010 and 2012. A total of 16 patients affected by unresectable stage III or IV melanoma received panobinostat, a potent HDACi that previously showed promising results among preclinical studies in melanoma samples and hematologic malignancies (Giles et al., 2006). Results from this phase I clinical trial were published in 2016: among 15 evaluable patients, four achieved stable disease, with an ORR of 0% and a disease control rate of 27%. Due to the absence of partial or complete response, and the heavy toxicity of study drug, the trial was stopped (Ibrahim et al., 2016).

## Other Genes

### TERT

*TERT* gene needs to be addressed separately from the other genes, since the most common mutations in *TERT* are localized in *TERT* promoter and have not been systematically investigated in most of the studies included in our dataset. *TERT* gene codes for a telomere reverse transcriptase catalytic subunit, the principal mechanism of telomere maintenance in cancer cells.

Most of human cancers re-activate telomerase (Kim et al., 1994). The first studies that identified cancer related *TERT* promoter mutations were performed in melanoma patients. The most common *TERT* promoter mutations are −57A/C, −124C/T, −146C/T, upstream the *TERT* gene ATG (Horn et al., 2013). From TCGA dataset of 9,127 patients and 31 cancer types emerged that 27% of all analyzed samples harbored one of these promoter mutations (Barthel et al., 2017), placing them among the most frequent cancer mutations (Lorbeer and Hockemeyer, 2020). In particular, 73 out 115 SKCM melanoma samples (frequency of 63.4%) harbored *TERT* promoter mutations (The Cancer Genome Atlas Network, 2015). Interestingly, WGS studies analyzed *TERT* promoter reporting a frequency of 81.2% in acral and cutaneous melanomas (Supplementary Figure S9) (Hayward et al., 2017).

Then, a discordancy on the mutational status between the primary and metastatic lesion was found in a cohort of 194 primary nodular melanomas matched with 72 loco-regional metastases, while TERT protein expression was found to correlate with reduced patient survival (Hugdahl et al., 2018). A frequent *TERT* promoter polymorphism at −245 was associated with an increased rate of metastasis in melanoma (Nagore et al., 2019) and inversely correlated with BRAF mutation (Bruno et al., 2018). Moreover, mutations of the *TERT* promoter are more frequent in fast-growing melanomas rather than slow-growing ones and this characteristic could be used to identify more aggressive tumors that might benefit from adjuvant therapy (Nagore et al., 2016). Recently, CNV was found in 61.5% of acral melanoma which is consistent with a previous study that reported rates from 44.9 to 75% (Liang et al., 2017; Yu et al., 2018, 2020). In our skin melanoma dataset, *TERT* coding mutations (3.9%) and CNVs (5.5%) are described in Figures 2, 3 and Supplementary Figure S8A. Specifically, the 42 *TERT* coding variants found in our dataset were all not-pathogenic variants in melanoma, underlying *TERT* as a polymorphic gene.

No clinical trials for *TERT* mutated patients or with TERT inhibitors are ongoing.

### MTOR

The *MTOR* gene encodes a protein belonging to the phosphatidylinositol kinase-related kinases family, which regulates cell growth, proliferation, motility and survival, protein synthesis and transcription (Wullschleger et al., 2006). mTOR is regulated and responds to growth factors, energy metabolites and/or levels of nutrients (Vogt, 2001). mTOR is the catalytic subunit of two different multiprotein complexes, mTORC1 (highly sensitive to rapamycin) and mTORC2 (relatively insensitive to rapamycin) with different functions. AKT regulates the mTORC1 complex by phosphorylating and inhibiting the TSC-2 gene (Tuberous Sclerosis 2), which is a GTP-ase activating protein (GAP) that binds to TSC-1 (Tuberin) forming a complex and blocking the G Rheb protein. The inhibition of TSC-2 allows the Rheb protein to accumulate in a GTP-bound state and to activate mTORC1. mTORC1 regulates several key steps of protein synthesis, controlling the expression of proteins that promote cell proliferation and survival. The mechanism controlling mTORC2 is not yet well known; the activation of this complex, however, is linked to the PI3K signaling (Pópulo et al., 2012). mTOR, as key protein of the PI3K/AKT pathway, acts both upstream and downstream of AKT, and aberrant mTOR activation of promotes survival and proliferation of tumor cells in several human cancers (Saxton and Sabatini, 2017; Chamcheu et al., 2019). Nonsynonymous *MTOR* mutations are present in about 3.61–12% of melanoma patients (The Cancer Genome Atlas Network, 2015; AACR Project Genie Consortium, 2017). In a Chinese cohort of 412 melanoma patients, nonsynonymous *MTOR* mutations were found by NGS analysis in acral, mucosal, cutaneous melanomas (with and without chronic sun-induced damage), and unknown primary subtypes, with a frequency of 11.0, 14.3, 3.4–6.7, and 11.1%, respectively (Kong et al., 2016). In this study, H1968Y, P2213S and S2215Y were established as gain-of-function mutations sensitive to specific inhibitors (Kong et al., 2016). Similarly to P2213S and S2215Y, the same authors found another gain-of-function mutation (H2189Y) that was sensitive in heterozygous at the AKT inhibitor (AZD5363) and the phosphoinositide 3-kinase inhibitor (LY294002), and in homozygous at the mTOR inhibitor (everolimus) and the AKT inhibitors (AZD5363) and (MK-2206 2HCL), and the phosphoinositide 3-kinase inhibitor (LY294002) (Wu et al., 2018). Interestingly, analyzing 31 candidate genes existing in either the PI3K or MAPK pathway in 105 metastatic melanoma patients, the researchers found two novel nonsynonymous mutations (R2443^∗^ and L552F) in *MTOR* gene (Shull et al., 2012).

In our dataset, mutations and CNVs in *MTOR* are found in 7.9 and 2.3% of the samples, respectively, as shown in Figures 2, 3 and Supplementary Figure S8B. However, among the 78 *MTOR* coding mutations found in our dataset, only five were pathogenic (6.4%; 2 E1799K, 2 S2215F, and 1 I2500F). Interestingly, these mutations have previously been identified as activating mutations in cancer patients using publicly available databases of cancer genome sequencing data (Grabiner et al., 2014). This study found 33 *MTOR* mutations that confer pathway activation, not by reducing sensitivity to mTOR inhibitors, but by altering the response of mTORC1 signaling pathway to nutrient deprivation (Grabiner et al., 2014).

Regarding melanoma therapy, MTOR may be targeted by selective inhibitors (Chamcheu et al., 2019). Rapamycin, a specific mTORC1 inhibitor, inhibits the cell growth and proliferation in several melanoma cell lines (Buscà et al., 1996; Molhoek et al., 2005; Karbowniczek et al., 2008). Two other rapamycin analogs, everolimus, and temsirolimus, also showed promising results in preclinical studies but failed the clinical experimentation in melanoma patients (Table 2).

Everolimus, as single agent, was tested in a phase II trial with 53 metastatic melanoma patients: the drug led to obtain just two clinical responses with a heavy toxicity profile and the study was terminated for futility (Vera Aguilera et al., 2018). A combination of everolimus with carboplatin and paclitaxel as I line treatment for metastatic melanoma was also tested in a phase II trial. Among 70 enrolled patients, just 12 showed objective responses, with a median PFS of 4 months and a median OS of 10 months (Hauke et al., 2013). Bevacizumab and everolimus were tested in a phase clinical II trial with 57 metastatic melanoma patients and led to a disease control rate of 70%. Unfortunately, median PFS and OS were just 4 month and 8.6 months, respectively (Hainsworth et al., 2010). Lastly, a recent study evaluated the combination of everolimus, bevacizumab, carboplatin and paclitaxel (CPB) in a randomized phase II trial. A total of 149 patients affected by stage IV melanoma were treated with CPB or with CPB plus everolimus: no differences in terms of PFS or OS were noted among the two groups, but everolimus strongly increased toxicity (McWilliams et al., 2018).

A new attempt to bring attention back to everolimus comes from a recently published phase I study, that evaluated the safety and the efficacy of vatalanib plus everolimus in patients with advanced solid tumors, including melanoma. Among 70 evaluable patients, nine achieved a partial response and 41 a stable disease, with best results obtained in neuroendocrine tumors, renal cancer, melanoma and NSCLC (Zhu et al., 2020).

Regarding the use of everolimus in mTOR mutated patients, only two clinical trials exist to date, to our knowledge. The first one is a phase II clinical trial on everolimus in cancer patients with *TSC1* and *TSC2* mutation or activating *MTOR* mutation. The second is a single-arm, open-labeled and single-centered study of everolimus in selective patients with metastatic melanoma and mutations (Kinase domain) of mTOR. Both studies are ongoing, and thus results are not yet available.

Temsirolimus was tested in combination with sorafenib in a phase I (Davies et al., 2012) and in a phase II clinical trials (Margolin et al., 2012), with disappointing results. Indeed, not only this combination failed to achieve any clinical response, but it induced a significant toxicity at higher dose levels. More encouraging results came from a phase II clinical trial with temsirolimus and bevacizumab: among 17 treated patients, three obtained a partial response and nine a stable disease, with best responses obtained in BRAF wild type population (Slingluff et al., 2013).

### TACC1

*TACC1* gene belongs to the *TACC* gene family that encodes centrosomal proteins that may play roles in microtubule regulation and spindle function, and, thus, may be an important driver of genomic instability in cancer cells (Anafi et al., 2000; Conte et al., 2003). Moreover, TACC1 proteins play an important role in transcriptional regulation (Gas41, thyroid hormone receptor, and retinoid acid receptor α) and mRNA processing (LSM7 and SmG) (Conte et al., 2002; Guyot et al., 2010). Many studies reported that TACC1 expression was modified in several cancers (Line et al., 2002; Rhodes et al., 2002; Nguyen et al., 2005; Ghayad et al., 2009). TACC1 has been also reported to stimulate the RAS and PI3K pathways playing an oncogenic role in tumor formation in the murine mammary gland (Cully et al., 2005). Finally, FGFR-TACC fusions, including FGFR1-TACC1, were also described in glioblastomas since 2012 (Di Stefano et al., 2015; Lasorella et al., 2017).

*TACC1* mutations were first reported in a WES study of 121 melanoma tumor/normal pairs reporting five novel melanoma candidate genes, including *TACC1*, with a frequency of 7% which was confirmed in a further similar study of 25 metastatic/germline pairs (Berger et al., 2012; Hodis et al., 2012) (Supplementary Figure S8C). Finally, in a recent study several key genes involved in melanomagenesis, including *TACC1*, in 115 human melanoma cell lines, 248 patient-derived xenografts, 31 cell lines derived from PDX, and 68 patient tumors, an overall frequency rate of 1.47% was reported (Garman et al., 2017). In conclusion, in our dataset *TACC1* mutations and CNVs have a frequency of 2.9 and 2.3%, as shown in Figures 2, 3 and Supplementary Figure S8C. No clinical trials are available.

### CNOT9

*CNOT9*, also known as *RQCD1*, encodes for the CCR4-NOT transcription complex subunit 9 that plays a role as transcription cofactor in multiple biological processes including cellular differentiation and RNA processing (Mathys et al., 2014). Several NGS studies revealed the presence at low frequency of recurrent mutations in *RQCD1/CNOT9* (P131L) in melanoma samples (Dutton-Regester et al., 2014). Interestingly, Wong SQ et al. found that melanoma tumors harboring *RQCD1/CNOT9* P131L mutation were associated with increased thickness, head and neck and upper limb location, lentigo maligna melanoma subtype, and *BRAF* V600K but not V600E or *NRAS* Q61 mutations (Wong et al., 2015). *CNOT9* alterations in our dataset are shown in Figures 2, 3 and Supplementary Figure S8D (frequency mutations and CNVs of 2.9 and 1.3%, respectively).

The functional oncogenic role of *CNOT9*/*RQCD1* in melanoma remains currently unknown although limited studies revealed RQCD1 implication in AKT activation and cell proliferation (Ajiro et al., 2009, 2010). In particular, the *CNOT9*/*RQCD1* mutant has demonstrated to stimulate stronger immune responses than the wild type, implicating the formation of a neoantigen that could be a potential therapeutic target although no drugs or clinical trials are available (Wong et al., 2015).

### YAP1

*YAP1* encodes for transcriptional coactivators belonging to the Hippo pathway. The core of the Hippo pathway in mammals consists of a kinase cascade, MST1/2 and LATS1/2, as well as downstream effectors, transcriptional coactivators YAP and TAZ (Ma et al., 2019). The Hippo pathway plays a crucial role in organ size control by regulation of cell proliferation, apoptosis, cell differentiation, and cell migration. Moreover, the upregulation of the Hippo pathway downstream effectors, such as YAP and TAZ, are common across various cancers since mutations and altered expression of its core components promote the migration, invasion, malignancy of cancer cells (Moroishi et al., 2015; Yu et al., 2015; Han, 2019). Very recently, 9,125 tumor samples were profiled revealing a widespread dysregulation of Hippo pathway components in multiple human cancer types, including melanoma (Sanchez-Vega et al., 2018). In particular, a strong interaction between Hippo pathway and *GNAQ* and *GNA11* oncogenes was observed. Several studies reported the functional role of YAP in uveal melanoma cells carrying *GNAQ/11* mutations (Feng et al., 2014; Yu et al., 2014). In uveal melanoma ATCG samples, only one among 80 uveal melanoma patients revealed a missense mutation in *YAP* (G369R) co-occurring with *GNAQ* R183Q and *GNA11* R183C (Bakhoum and Esmaeli, 2019). Moreover, no *YAP* CNV was shown in uveal melanoma samples by a comprehensive molecular characterization of the Hippo core genes in 9,125 tumor samples (Wang Y. et al., 2018). In our skin melanoma dataset, *YAP1* alterations are reported in Figures 2, 3 and Supplementary Figure S10, with an overall frequency mutation and CNVs of 1.4 and 2.6%, respectively.

Although the central role of YAP in uveal melanomagenesis is deeply investigated, the role of Hippo/YAP signaling in cutaneous melanoma is less understood. Zhang et al. (2019) proved for the first time that YAP hyperactivity is elevated in invasive melanoma cell lines, induce invasion in normally non-invasive melanoma cells, induces spontaneous melanoma metastasis *in vivo* and promotes melanoma cell invasion by regulating expression of AXL, CYR61 and CRIM1. Moreover, they did not found association between YAP activity and the *BRAF/NRAS* mutation status. Of note, they found for the first time somatic activating mutations in *YAP* gene by WES. Indeed, they identified seven independent serine to alanine substitutions in YAP gene (called YAP-7SA) from a primary cutaneous melanoma patient carrying *BRAF* V600E and *RAC1* P29S mutation. In vitro study confirmed that YAP-7SA encodes a hyperactive version of the YAP protein (Zhang et al., 2019). Despite the great interest of scientific community toward the role of Hippo pathway in cancer, no drugs and clinical trials direct to YAP in melanoma are described.

## Conclusion and Perspectives

The better definition of the genetic mechanisms underlying melanomagenesis allowed to synthesize new molecular targeted drugs that radically changed the prognosis of melanoma patients.

In this review we analyzed more than 30 genes other than *BRAF*, that can be or become targets for new molecular target therapies. We collected mutation data on a total of 992 melanoma samples analyzed by WES or WGS to prove the genetic heterogeneity of melanoma, beyond the *BRAF* mutation. Only 11 of them (1.1%) showed no coding mutations, whereas 298 (30%) showed a mutation in one of the *RAS* genes (mostly *NRAS*) and most of the samples studied (698, 70%) showed mutations in non-*RAS* genes (Figures 2, 3) suggesting potential new roads/candidates for targeted therapy.

We also provide an overview of the related clinical trials, ongoing or completed, to better outline the state of molecular – derived clinical research (Table 2). Among the 33 selected established and candidate melanoma driver genes here described, clinical trials have been performed for 11 genes (33.3%) and this percentage could be increased if we considered ongoing trials with immunotherapy although it was not the topic of our review. Moreover, several preclinical studies are showing that some drugs could have anti-tumor ability toward melanoma driver genes opening future clinical studies and new potential therapeutic perspectives.

In this scenario, we strongly believe that in a near future, the use of NGS could extend the cohort of patients that could benefit by this therapeutic approach. Indeed, at least two clinical trials including melanoma patients (NCT02465060 and NCT02645149) are currently ongoing to prove this concept, enrolling hundreds of patients regardless of their neoplasm, and treating them with the specific drug targeting the specific identified mutation. Hopefully, the results of these experimentations will be a milestone in medical oncology because will allow us to put into practice a real personalized therapy, tailored on what is written in tumor genome.

## Author Contributions

IV and ET contributed equally in conceiving the review focus, conducting the literature review and summarizing the manuscript, analyzed data, wrote the first draft, and finalized the manuscript. PG designed and coordinated the review. BD, LP, VA, WB, AB, FS, and PG reviewed the literature, revised and made corrections to the manuscript. All authors have read and agreed to the final version of the manuscript.

## Conflict of Interest

The authors declare that the research was conducted in the absence of any commercial or financial relationships that could be construed as a potential conflict of interest.

## Footnotes

1 https://www.mycancergenome.org/

## Supplementary Material

FIGURE S1 *NRAS* **(A)**, *KRAS* **(B)**, *HRAS* **(C)**, and *PREX2* **(D)** mutations and CNV frequency in the 10 NGS selected studies. Mutations are indicated in green and CNV in purple. Only four studies with available CNV information were considered for CNV analysis.

FIGURE S2 *TP53* **(A)**, *NF1* **(B)**, *ARID2* **(C)**, and *CDKN2A* **(D)** mutations and CNV frequency in the 10 NGS selected studies. Mutations are indicated in green and CNV in purple. Only four studies with available CNV information were considered for CNV analysis.

FIGURE S3 *CDK4* **(A)**, *PTEN* **(B)**, *PPP6C* **(C)**, and *CTNNB1* **(D)** mutations and CNV frequency in the 10 NGS selected studies. Mutations are indicated in green and CNV in purple. Only four studies with available CNV information were considered for CNV analysis.

FIGURE S4 *DDX3X* **(A)**, *RASA2* **(B)**, *SF3B1* **(C)**, and *RAC1* **(D)** mutations and CNV frequency in the 10 NGS selected studies Mutations are indicated in green and CNV in purple. Only four studies with available CNV information were considered for CNV analysis.

FIGURE S5 *MAP2K1* **(A)**, *MAP2K2* **(B)**, *KIT* **(C)**, and *RB1* **(D)** mutations and CNV frequency in the 10 NGS selected studies. Mutations are indicated in green and CNV in purple. Only four studies with available CNV information were considered for CNV analysis.

FIGURE S6 *FBXW7* **(A)**, *PIK3CA* **(B)**, *EZH2* **(C)**, and *WT1* **(D)** mutations and CNV frequency in the 10 NGS selected studies. Mutations are indicated in green and CNV in purple. Only four studies with available CNV information were considered for CNV analysis.

FIGURE S7 *SNX31* **(A)**, *IDH1* **(B)**, *STK19* **(C)**, and *MITF* **(D)** mutations and CNV frequency in the 10 NGS selected studies. Mutations are indicated in green and CNV in purple. Only four studies with available CNV information were considered for CNV analysis.

FIGURE S8 *TERT* **(A)**, *MTOR* **(B)**, *TACC1* **(C)**, and *CNOT9* **(D)** mutations and CNV frequency in the 10 NGS selected studies. Mutations are indicated in green and CNV in purple. Only four studies with available CNV information were considered for CNV analysis.

FIGURE S9 *TERT* promoter mutations in 160 skin melanomas. *TERT* promoter mutations were derived by 129 cutaneous melanoma and 31 acral melanomas (Hayward et al., 2017).

FIGURE S10 *YAP1* mutations and CNV frequency in the 10 NGS selected studies. Mutations are indicated in green and CNV in purple. Only four studies with available CNV information were considered for CNV analysis.
