# Supplementary figures and images for: Non-BRAF Mutant Melanoma: Molecular Features and Therapeutical Implications
Source: Front Mol Biosci. 2020 Jul 24;7:172. doi: 10.3389/fmolb.2020.00172 (PMC7396525; doi:10.3389/fmolb.2020.00172)

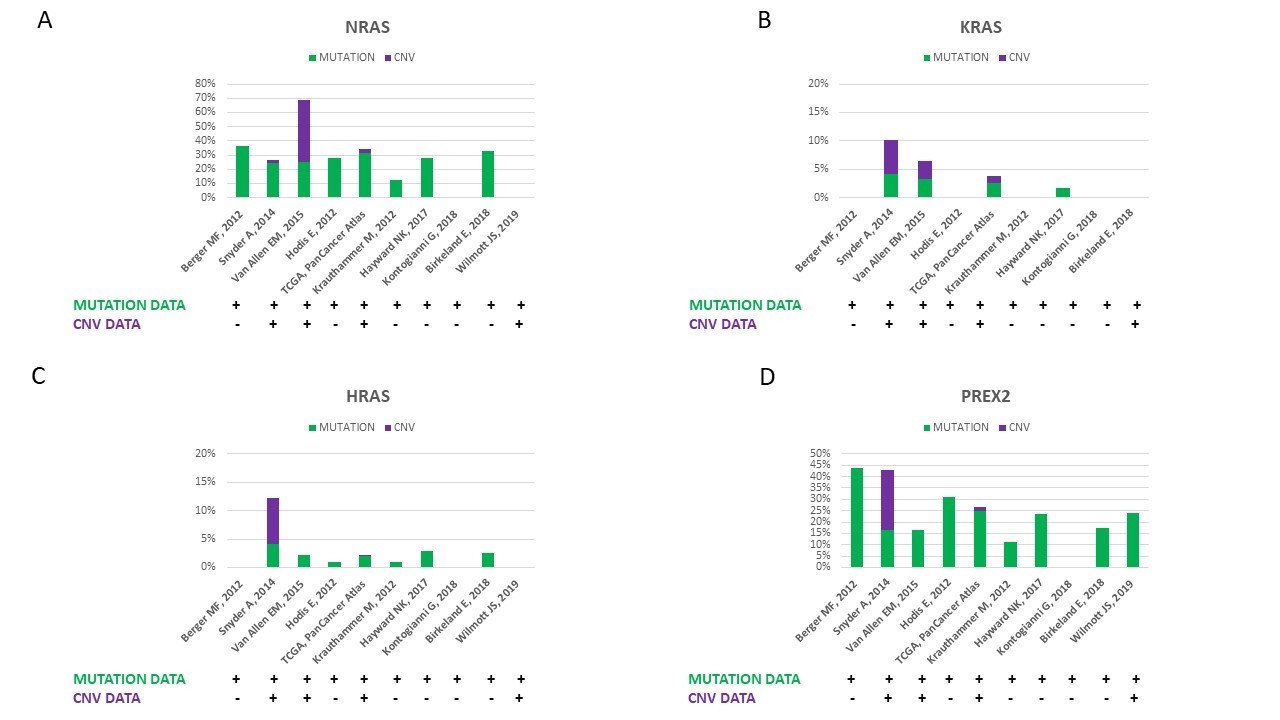

Supplement: FIGURE S1 — NRAS (A), KRAS (B), HRAS (C), and PREX2 (D) mutations and CNV frequency in the 10 NGS selected studies. Mutations are indicated in green and CNV in purple. Only four studies with available CNV information were considered for CNV analysis. [file Image_1.JPEG]

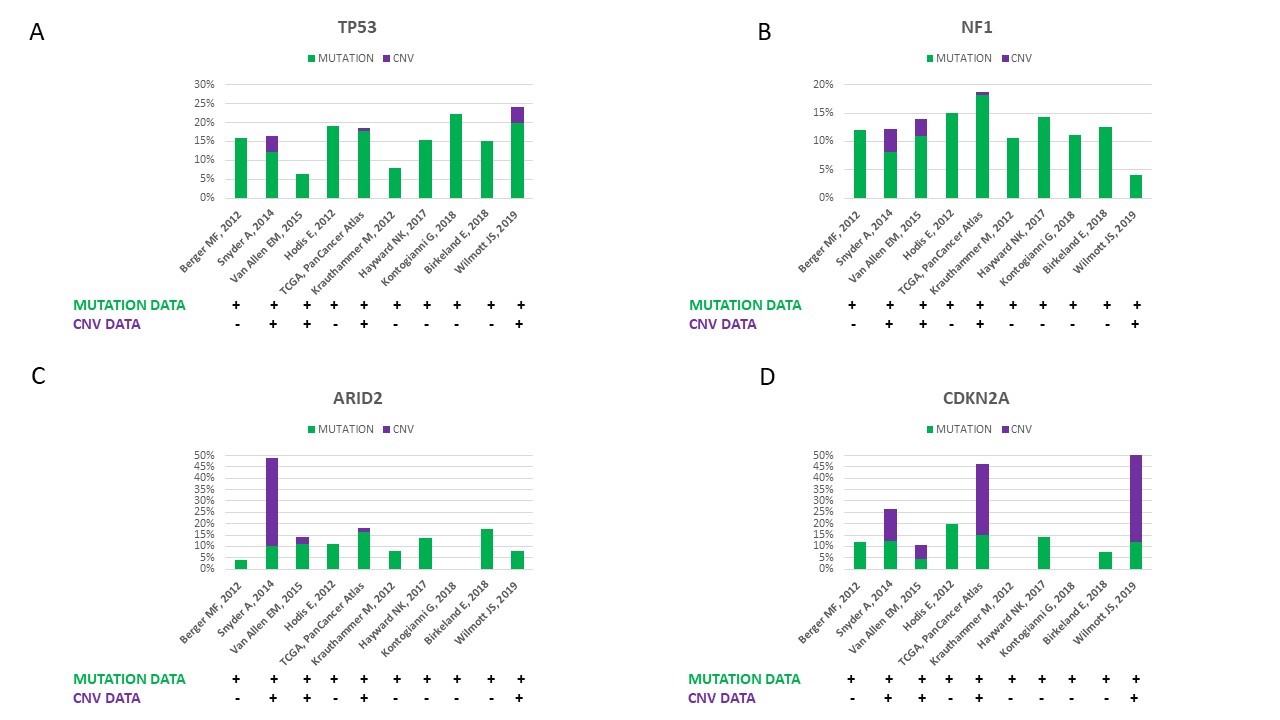

Supplement: FIGURE S2 — TP53 (A), NF1 (B), ARID2 (C), and CDKN2A (D) mutations and CNV frequency in the 10 NGS selected studies. Mutations are indicated in green and CNV in purple. Only four studies with available CNV information were considered for CNV analysis. [file Image_2.JPEG]

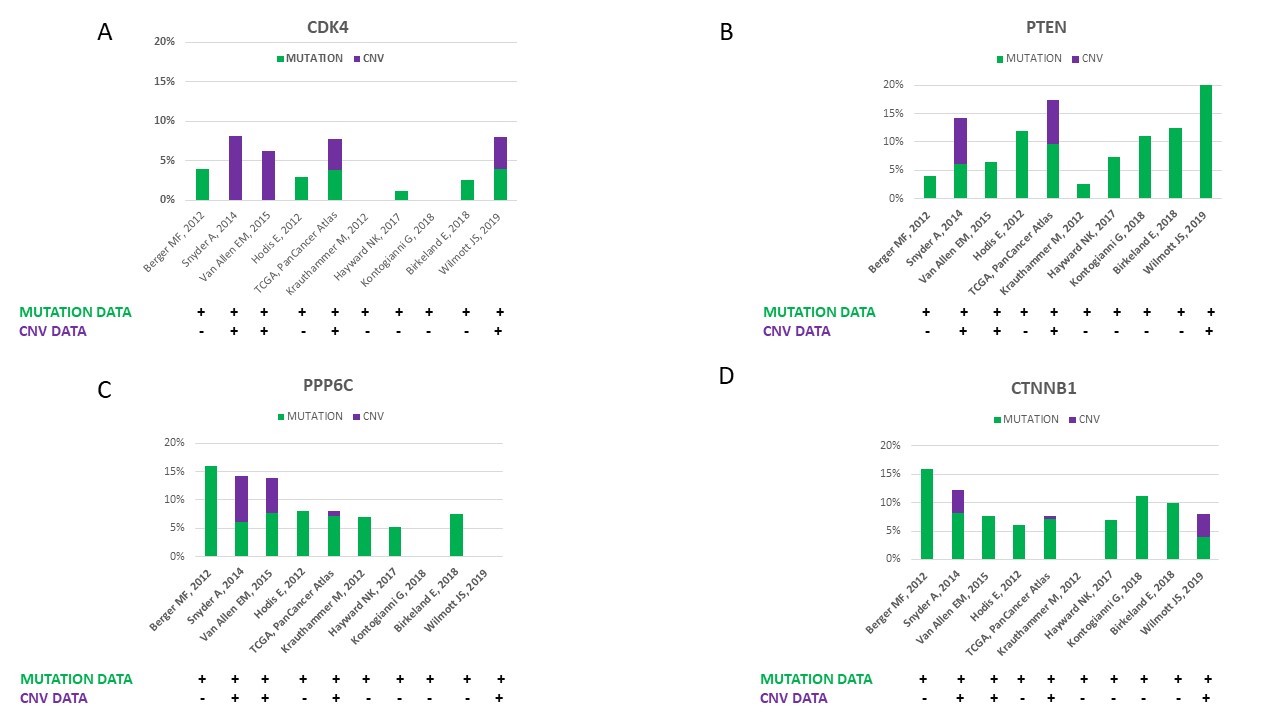

Supplement: FIGURE S3 — CDK4 (A), PTEN (B), PPP6C (C), and CTNNB1 (D) mutations and CNV frequency in the 10 NGS selected studies. Mutations are indicated in green and CNV in purple. Only four studies with available CNV information were considered for CNV analysis. [file Image_3.JPEG]

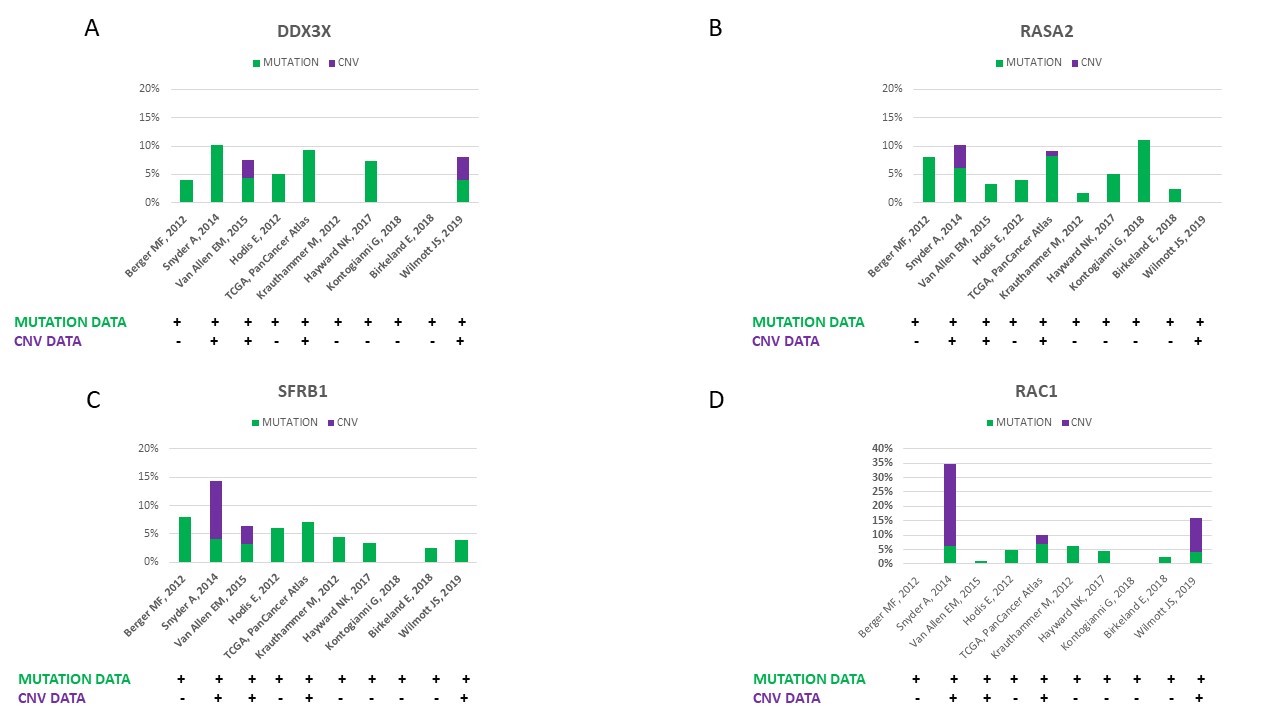

Supplement: FIGURE S4 — DDX3X (A), RASA2 (B), SF3B1 (C), and RAC1 (D) mutations and CNV frequency in the 10 NGS selected studies Mutations are indicated in green and CNV in purple. Only four studies with available CNV information were considered for CNV analysis. [file Image_4.JPEG]

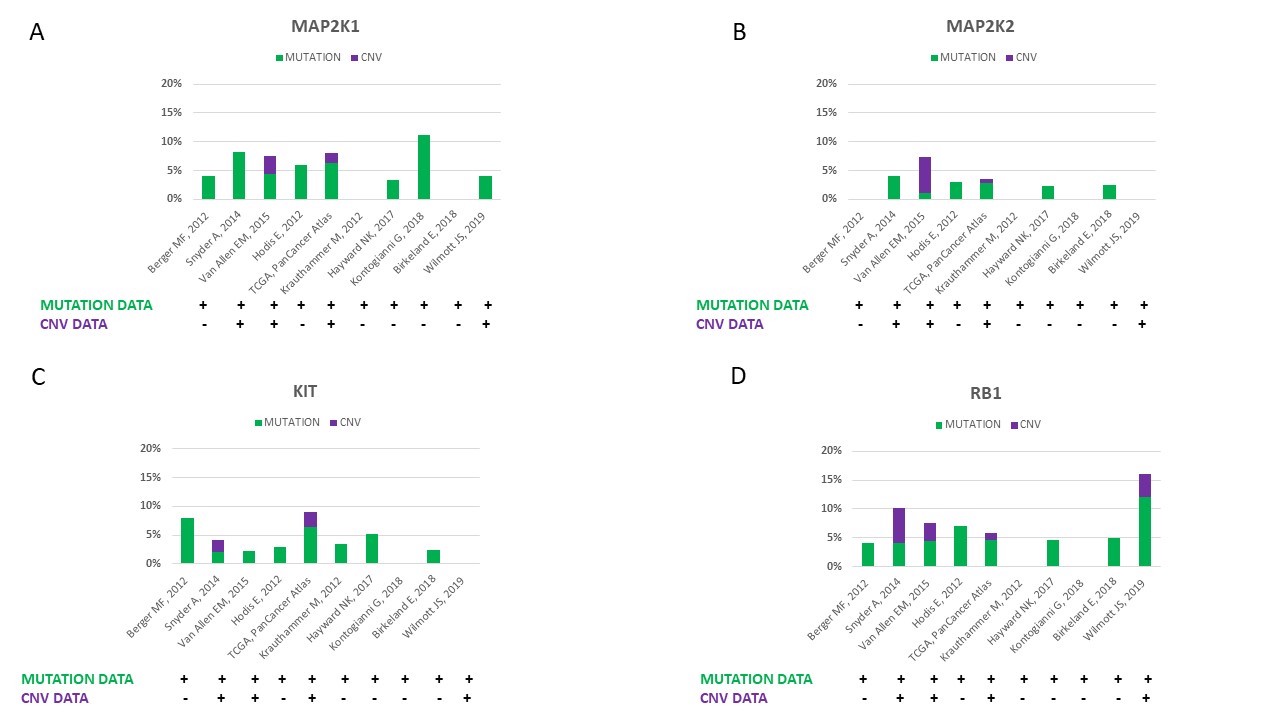

Supplement: FIGURE S5 — MAP2K1 (A), MAP2K2 (B), KIT (C), and RB1 (D) mutations and CNV frequency in the 10 NGS selected studies. Mutations are indicated in green and CNV in purple. Only four studies with available CNV information were considered for CNV analysis. [file Image_5.JPEG]

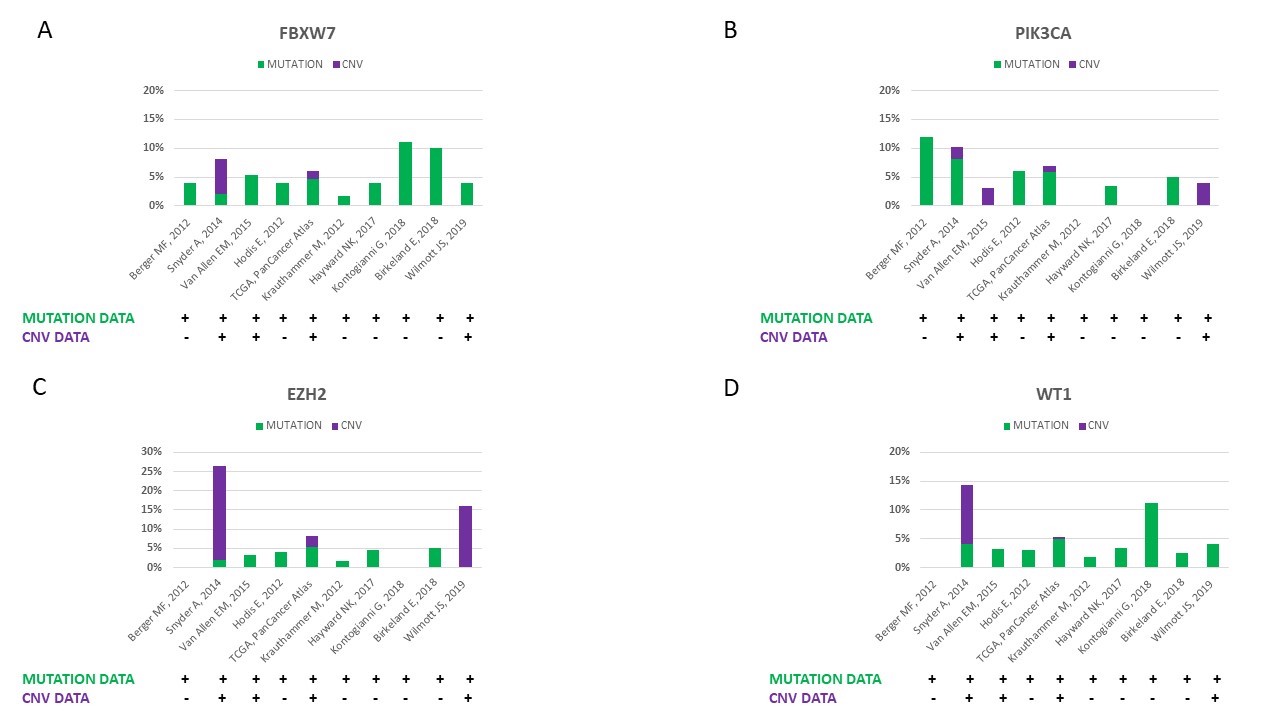

Supplement: FIGURE S6 — FBXW7 (A), PIK3CA (B), EZH2 (C), and WT1 (D) mutations and CNV frequency in the 10 NGS selected studies. Mutations are indicated in green and CNV in purple. Only four studies with available CNV information were considered for CNV analysis. [file Image_6.JPEG]

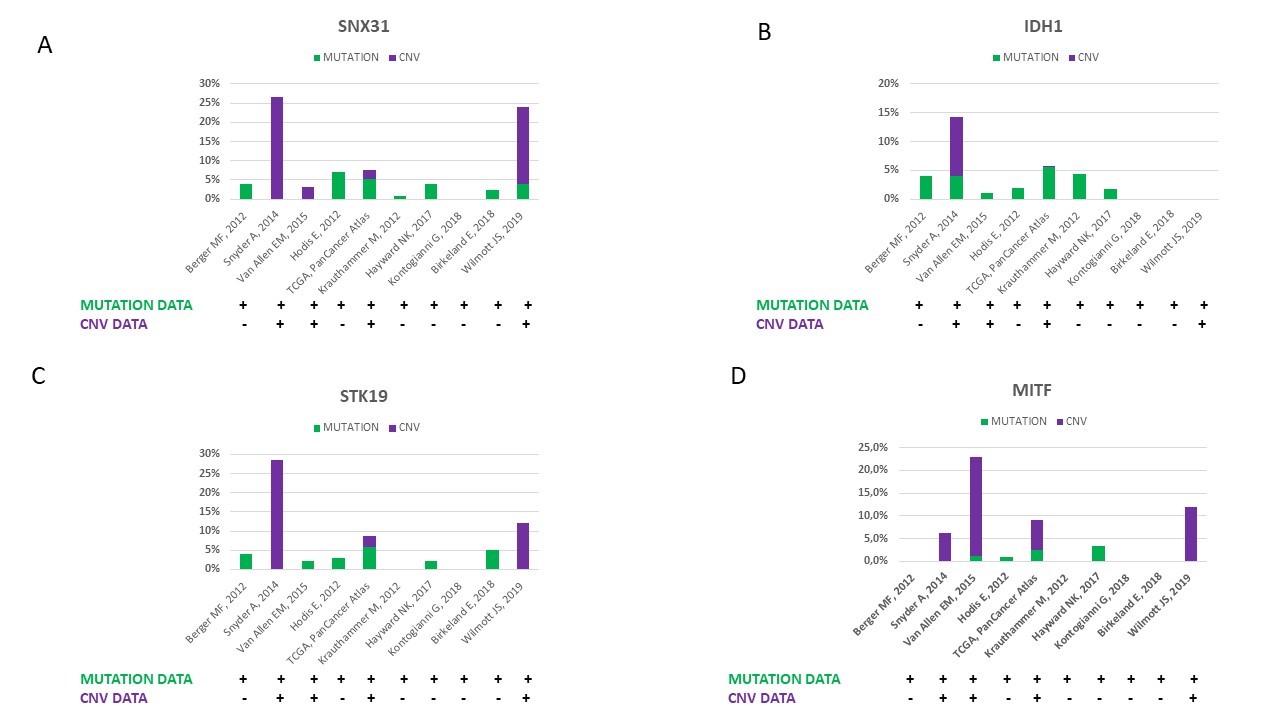

Supplement: FIGURE S7 — SNX31 (A), IDH1 (B), STK19 (C), and MITF (D) mutations and CNV frequency in the 10 NGS selected studies. Mutations are indicated in green and CNV in purple. Only four studies with available CNV information were considered for CNV analysis. [file Image_7.JPEG]

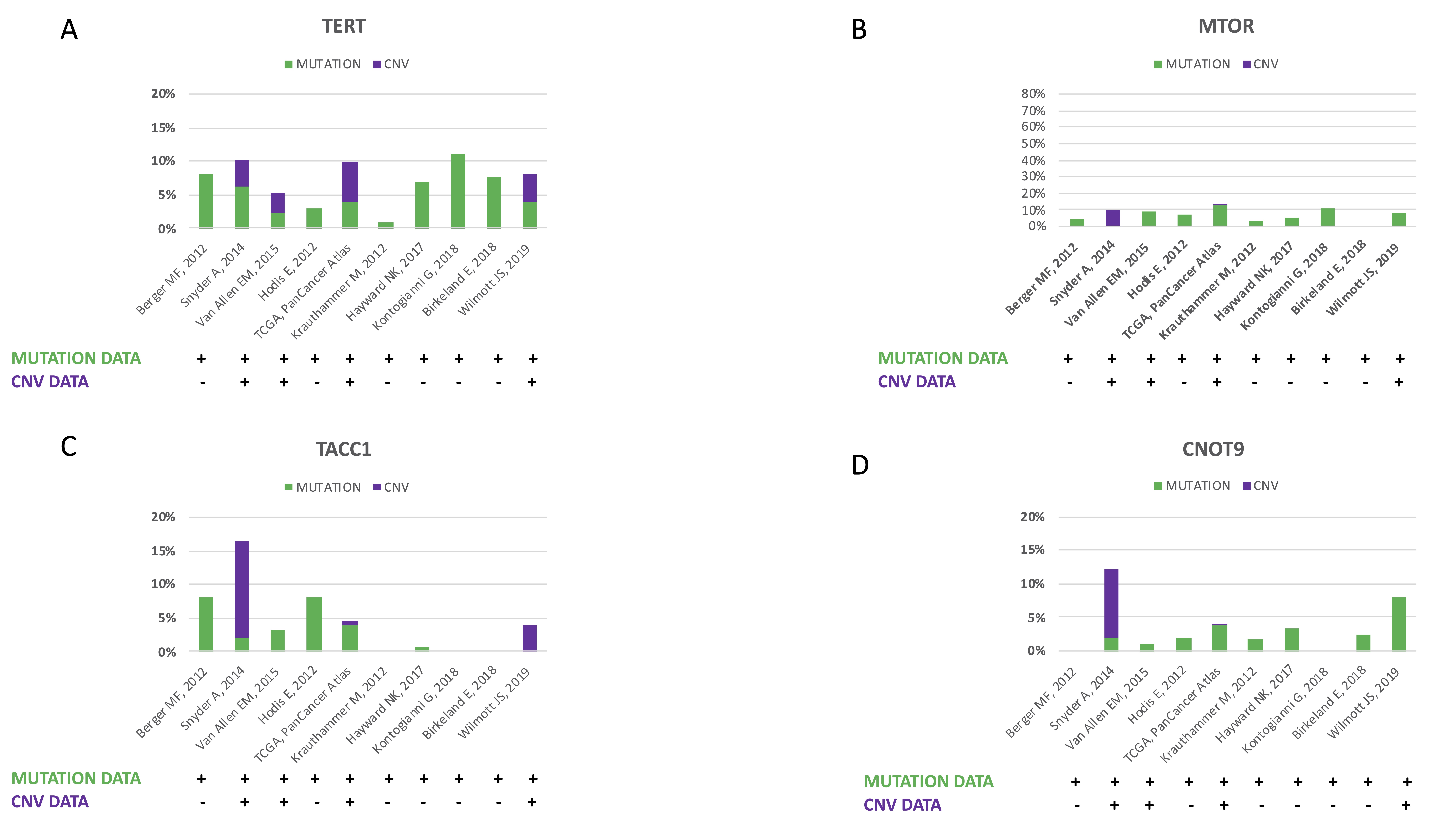

Supplement: FIGURE S8 — TERT (A), MTOR (B), TACC1 (C), and CNOT9 (D) mutations and CNV frequency in the 10 NGS selected studies. Mutations are indicated in green and CNV in purple. Only four studies with available CNV information were considered for CNV analysis. [file Image_8.JPEG]

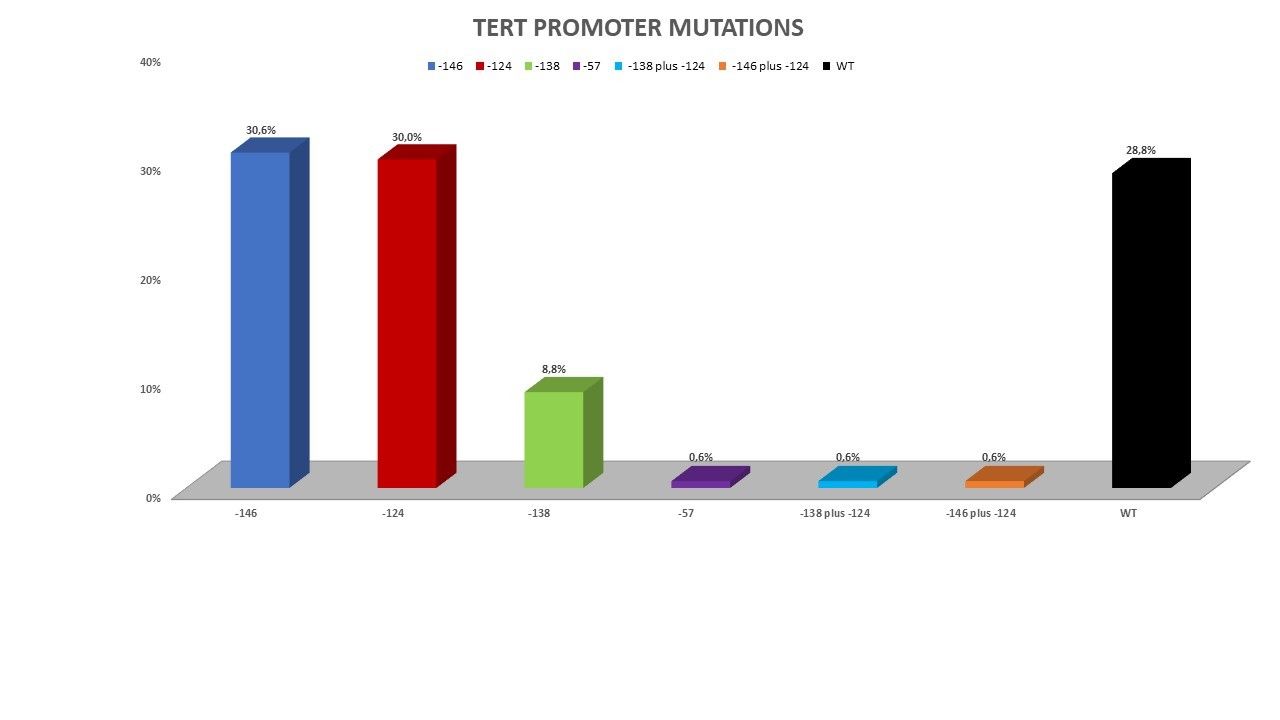

Supplement: FIGURE S9 — TERT promoter mutations in 160 skin melanomas. TERT promoter mutations were derived by 129 cutaneous melanoma and 31 acral melanomas (Hayward et al., 2017). [file Image_9.JPEG]

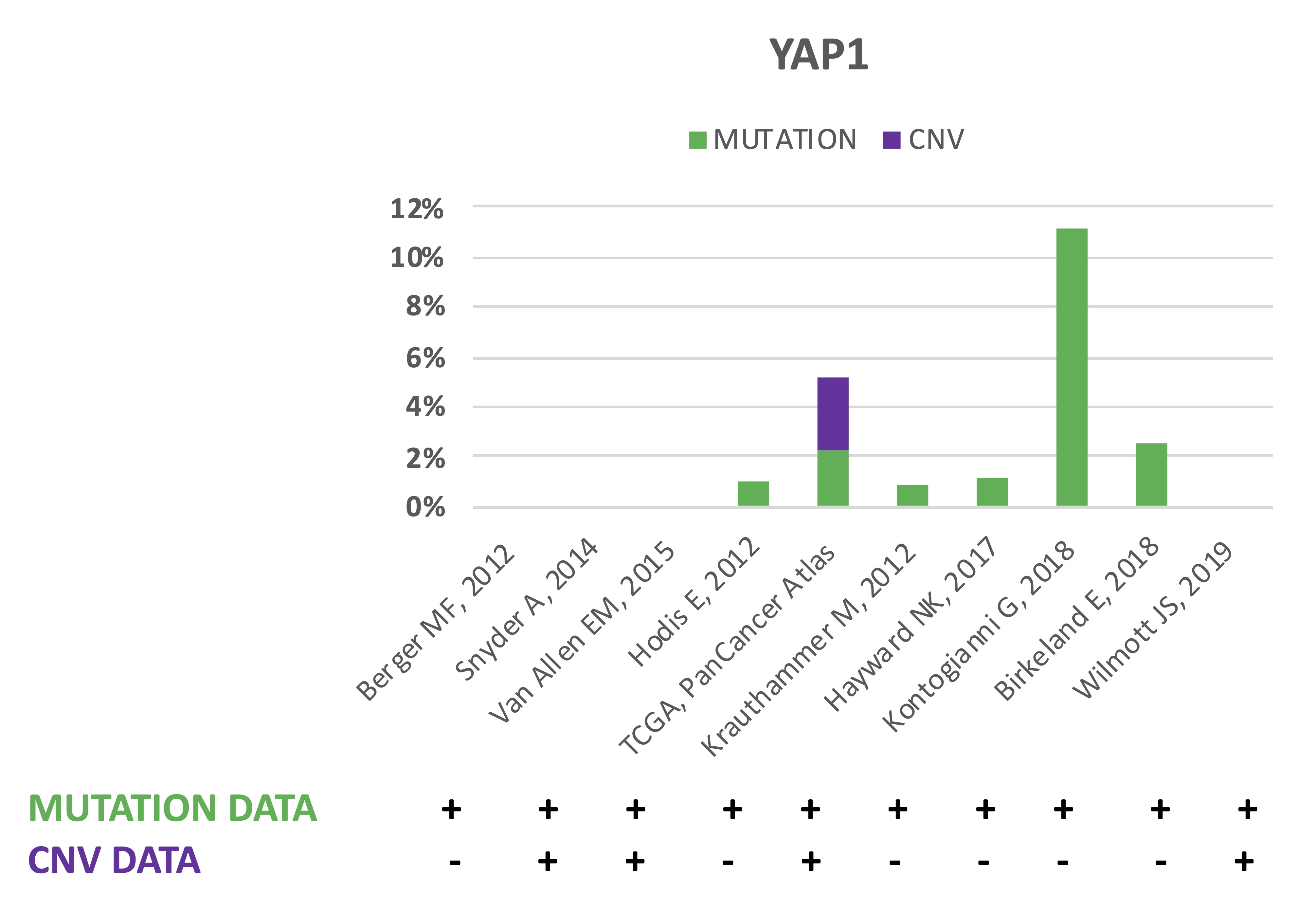

Supplement: FIGURE S10 — YAP1 mutations and CNV frequency in the 10 NGS selected studies. Mutations are indicated in green and CNV in purple. Only four studies with available CNV information were considered for CNV analysis. [file Image_10.JPEG]
